# Kinetochore-Dependent Microtubule Rescue Ensures Their Efficient and Sustained Interactions in Early Mitosis

**DOI:** 10.1016/j.devcel.2011.09.006

**Published:** 2011-11-15

**Authors:** Sapan R. Gandhi, Marek Gierliński, Akihisa Mino, Kozo Tanaka, Etsushi Kitamura, Lesley Clayton, Tomoyuki U. Tanaka

**Affiliations:** 1 Wellcome Trust Centre for Gene Regulation & Expression, College of Life Sciences, University of Dundee, Dundee DD1 5EH, UK; 2 Data Analysis Group, College of Life Sciences, University of Dundee, Dundee DD1 5EH, UK

## Abstract

How kinetochores regulate microtubule dynamics to ensure proper kinetochore-microtubule interactions is unknown. Here, we studied this during early mitosis in *Saccharomyces cerevisiae*. When a microtubule shrinks and its plus end reaches a kinetochore bound to its lateral surface, the microtubule end attempts to tether the kinetochore. This process often fails and, responding to this failure, microtubule rescue (conversion from shrinkage to growth) occurs, preventing kinetochore detachment from the microtubule end. This rescue is promoted by Stu2 transfer (ortholog of vertebrate XMAP215/ch-TOG) from the kinetochore to the microtubule end. Meanwhile, microtubule rescue distal to the kinetochore is also promoted by Stu2, which is transported by a kinesin-8 motor Kip3 along the microtubule from the kinetochore. Microtubule extension following rescue facilitates interaction with other widely scattered kinetochores, diminishing long delays in collecting the complete set of kinetochores by microtubules. Thus, kinetochore-dependent microtubule rescue ensures efficient and sustained kinetochore-microtubule interactions in early mitosis.

## Graphical abstract

**Graphical Abstract:**

**Figure:**
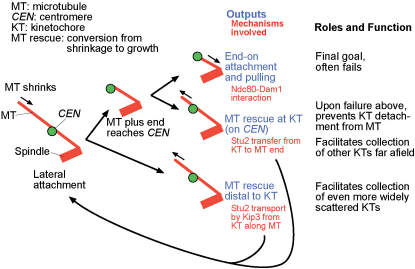

**Highlights:**

► Microtubule (MT) rescue is promoted by a laterally associated kinetochore (KT) ► MT rescue at and distal to the KT is promoted by Stu2 transfer and transport from the KT ► MT rescue prevents KT detachment from the MT end when KT end-on tethering fails ► MT extension following rescue facilitates collection of other widely scattered KTs

## Introduction

For proper chromosome segregation in mitosis, kinetochores (KTs) must establish correct interactions with spindle microtubules (MTs) during early mitosis (prometaphase) (Tanaka, 2010). A KT initially interacts with the lateral surface of a MT extending from a spindle pole (spindle-pole MT) (lateral KT-MT attachment; Rieder and Alexander, 1990; Tanaka et al., 2005). Once contact is made, the KT is transported along the MT toward a spindle pole. Subsequently, the KT is tethered at the MT plus end and pulled further as the MT shrinks (end-on KT-MT attachment and end-on pulling) (Rieder and Alexander, 1990; Tanaka et al., 2007; Tanaka, 2010). The conversion from lateral to end-on attachment is crucial because end-on attachment is more robust (Grishchuk et al., 2005; Joglekar et al., 2010; Tanaka et al., 2007) and thought to be necessary to sustain KT-MT attachment when tension is applied across sister KTs upon their biorientation, i.e., their attachment to MTs from the opposite poles (Maure et al., 2011; Tanaka, 2010). During such stepwise development of the KT-MT interactions, MTs regulate the motion and localization of KTs. To ensure this process, the KT may also need to regulate the dynamics of its associated MT; however, such regulation largely remains elusive.

The initial KT-MT interaction has been recently analyzed in the budding yeast *Saccharomyces cerevisiae*. In this organism, spindle-pole bodies (SPBs: equivalent to centrosomes in metazoan cells) are embedded in the nuclear envelope, which remains intact during mitosis. KTs are connected to SPBs by MTs through almost the entire cell cycle (Winey and O'Toole, 2001). However, KTs are disassembled upon centromere DNA replication during early S phase, making centromeres detach from MTs and move away from a spindle pole (Kitamura et al., 2007). Subsequently, the KT is reassembled and, similarly to vertebrate cells, interacts with the lateral surface of a spindle-pole MT. The KT subsequently moves along the MT toward a spindle pole (sliding); however, this speed is slower than the MT depolymerization speed and the MT plus end often catches up with the KT, leading to the establishment of end-on attachment (Tanaka et al., 2007). These processes can be analyzed with higher spatial resolution using an engineered assay system (Tanaka et al., 2005).

During lateral KT-MT attachment, the KT changes the dynamics of its associated MT from shrinkage to growth, i.e., converts tubulin depolymerization to polymerization at the MT plus end (MT rescue) (Tanaka et al., 2005). As a result, the period of lateral KT-MT attachment is extended and conversion to the end-on attachment seems to be delayed. Perplexingly, if end-on attachment is a more mature form of KT-MT interaction, why do cells delay conversion to end-on attachment? Does KT-dependent MT rescue bring any benefit to KT-MT interaction?

Our previous study revealed that KT-dependent MT rescue often happens before the plus end of a shrinking MT catches up with a KT that is sliding poleward along a MT (Tanaka et al., 2005). MT rescue distal to the KT is accompanied by the transport of Stu2 from the KT to the MT plus end. Stu2 belongs to an evolutionarily conserved family of MT-binding proteins, which includes XMAP215 in *Xenopus* and ch-TOG in mammals (Gard et al., 2004; Ohkura et al., 2001; Wang and Huffaker, 1997). Stu2 family proteins possess repeats, called TOG domains, at their N-termini, which directly bind tubulin dimers (Al-Bassam et al., 2006; Spittle et al., 2000) and can promote their polymerization at the MT plus end (Brouhard et al., 2008). However, Stu2 family proteins cannot move directionally along MTs by themselves (Brouhard et al., 2008) and it is still unclear how Stu2 is transported from the KT along a MT. Here, we studied the mechanism of KT-dependent MT rescue and its benefits for KT-MT interactions during early mitosis in budding yeast.

## Results

### A Microtubule Carrying a Kinetochore on Its Lateral Side Is Rescued Either before or after Its Plus End Catches Up with the Kinetochore

**Figure 1:**
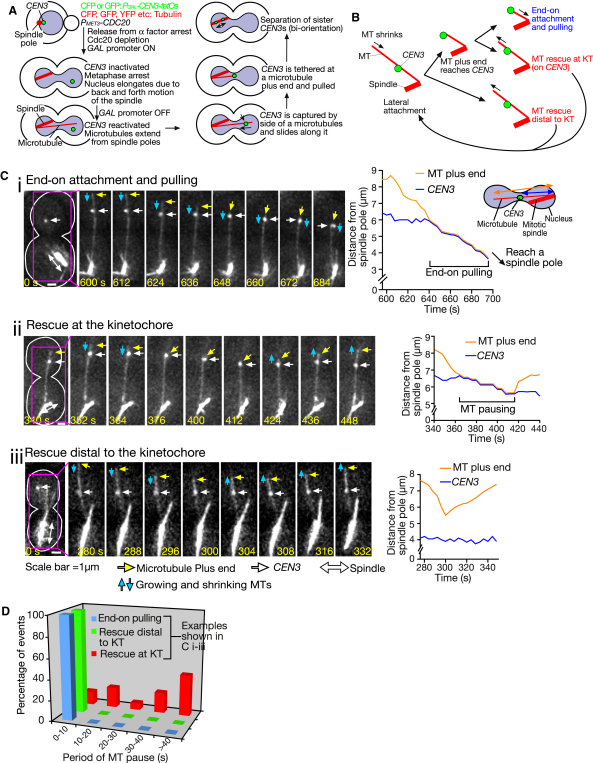
A Shrinking Microtubule, Whose Lateral Surface Is Associated with a Kinetochore, Shows Rescue before or after Its Plus End Reaches the Kinetochore (A) Experimental system to analyze the initial interaction between a KT (on *CEN3*) and an individual spindle-pole MT (Tanaka et al., 2005). (B) Schematic diagram showing possible fates of a shrinking MT: when a MT plus end catches up with *CEN3* on its lateral side, either end-on attachment/pulling of *CEN3* occurs, or the MT is rescued at *CEN3* (rescue at the KT). MT rescue also happens before the plus end reaches *CEN3* (rescue distal to the KT). Once end-on pulling starts, no MT rescue occurs. (C) Time-lapse images of *P_MET3_*-*CDC20 P_GAL_-CEN3-tetOs TetR*-*GFP GFP*-*TUB1* (T7448) cells. The cells were treated with α factor in methionine drop-out medium with 2% raffinose for 2.5 hr, and then released to YP medium containing 2% galactose, 2% raffinose, and 2 mM methionine. After 3 hr, cells were suspended in synthetic complete medium containing 2% glucose and 2 mM methionine. Images were collected every 4 s for 20 min, using the GFP channel. Zero time is set arbitrarily for the first panel, in which the cell shape is outlined in white. Scale bar, 1 μm. Each example shows (i) end-on attachment/pulling, (ii) MT rescue at the KT (on *CEN3*), and (iii) rescue distal to the KT. Graphs show MT length (orange) and *CEN3*–spindle-pole distance (blue) in the cells. (D) The MT plus ends stay with the KT (on *CEN3*) for an appreciable time prior to rescue, during which the MT shows pausing. Time-lapse images were acquired as in (C). Graph shows the period of MT pausing (shrinkage <1.0 μm/min and no growth), prior to end-on attachment/pulling (1.5–2.0 μm/min; n = 13), MT rescue at the KT (n = 13), and distal to the KT (n = 7).

Lateral and end-on KT-MT attachments (see Introduction) found in physiological conditions (Kitamura et al., 2007) can be recapitulated in an engineered assay system, which allows more detailed observation of individual KT-MT interactions (Tanaka et al., 2005, 2007). We used this assay, in which KT assembly was delayed on a particular centromere (*CEN3*) by transcription from an adjacently inserted promoter (Figure 1A). This procedure increased the distance between *CEN3* and the mitotic spindle. While cells were arrested in metaphase, we reactivated *CEN3*, leading to KT reassembly and interaction with the lateral surface of a single spindle-pole MT (Tanaka et al., 2005).

During lateral attachment, a spindle-pole MT shrank and its plus end often caught up with *CEN3*. In ∼40% of such cases, end-on attachment was established and followed by end-on pulling of *CEN3* (Figures 1B and 1Ci). However, in the remaining ∼60% cases, the MT was rescued, i.e., showed conversion from shrinkage to growth (rescue at the KT) (Figures 1B and 1Cii). Intriguingly, rescue of a *CEN3*-associated MT also occurred even before its plus end reached *CEN3* (rescue distal to the KT) (Figures 1B and 1Ciii). The frequency of rescue at and distal to the KT was 64% and 36% (16 and 9 out of 25) of the total rescue events, respectively. Both types of MT rescue were dependent on the MT-associated KT (on *CEN3*); indeed, no rescue occurred if MTs were not associated with a KT (Tanaka et al., 2005). Furthermore, once end-on pulling of *CEN3* had started, no rescue was observed (Tanaka et al., 2007).

**Figure 2:**
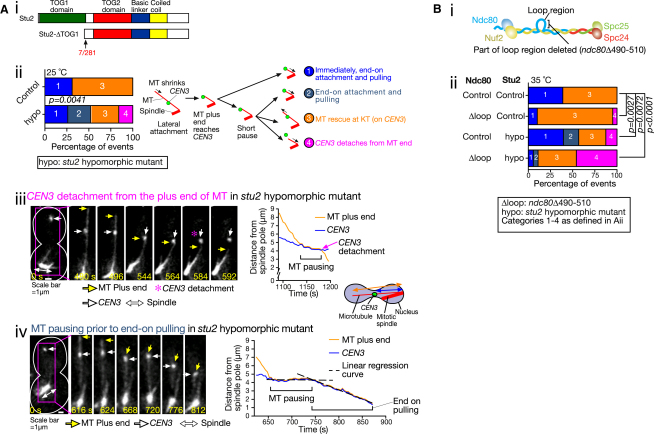
Microtubule Rescue at the Kinetochore Is Facilitated by Stu2 and Prevents a Loss of Kinetochore-Microtubule Attachment (A) Stu2 facilitates MT rescue at the KT and prevents loss of KT-MT attachment. *P_STU2_-stu2ΔTOG1* (T9323; *stu2* hypomorphic mutant [hypo]) and *P_STU2_-STU2^+^*(T9345; control) cells (i.e., with an extra *stu2* or *STU2^+^* gene at an auxotroph marker locus) with *P_MET3_*-*CDC20 P_GAL_-CEN3-lacOs TetR*-*GFP GFP*-*TUB1 STU2^+^* were treated, and their images were acquired as in Figure 1C. (i) Schematic diagrams show wild-type *STU2* and a mutant *stu2* with the TOG1 domain deleted. 7/281: 7–281 amino acid residues are deleted. (ii) Graph showing the proportion of the events (shown in the diagram) after the plus end of a shrinking MT catches up with *CEN3* in the control (n = 20) and *stu2* hypomorphic mutant (n = 38). Representative examples of (iii) *CEN3* detachment from the MT end (preceded by MT pausing) and (iv) end-on attachment/pulling (preceded by MT pausing), found in the *stu2* hypomorphic mutant. Zero time is set arbitrarily for the first panel. Scale bar, 1 μm. The graphs show MT length (orange) and *CEN3*–spindle-pole distance (blue). (B) The double mutant of *stu2* hypomorph and the *ndc80* loop region shows frequent *CEN3* detachment from the MT end. (i) Diagram showing the deletion in *ndc80Δ490–510* (Maure et al., 2011). (ii) *NDC80^+^ P_STU2_-STU2^+^* (T9460), *ndc80Δ490–510 P_STU2_-STU2^+^* (T9456), *NDC80^+^ P_STU2_-stu2ΔTOG1* (T9470), and *ndc80Δ490–510 P_STU2_-stu2ΔTOG1* (T9455) cells with *P_MET3_*-*CDC20 P_GAL_-CEN3-tetOs TetR*-*GFP Venus*-*TUB1 STU2^+^* were treated, and their images were acquired as in Figure 1C, except that the temperature for cell culture was shifted from 25°C to 35°C, 30 min prior to image acquisition. The restrictive temperature for *ndc80Δ490–510* is 35°C (Maure et al., 2011). Graph shows the proportion of the events (categorized as in Aii, diagram) after the plus end of a shrinking MT catches up with *CEN3* (n = 31, 33, 23, and 39 from top to bottom). Ndc80 control was Ndc80 wild-type. Stu2 hypo and control were as defined in (A). See also Figure S1.

Establishment of end-on attachment (which is verified by subsequent end-on pulling) is probably crucial for subsequent sister KT biorientation (Maure et al., 2011; Tanaka, 2010). However, if end-on attachment is the essential next step for KT-MT interaction, what are the benefits of KT-dependent MT rescue, which extends the duration of lateral attachment and seems to delay establishment of end-on attachment? A clue to this question was obtained regarding MT rescue at the KT, when we recently studied mutants of the Ndc80 loop region (Maure et al., 2011), a distinct motif looping out from the coiled-coil shaft of the Ndc80 complex (see Figure 2Bi). These mutants were defective in forming end-on attachment; indeed, when the MT plus end caught up with a laterally associated KT, end-on attachment was rarely established. Given this, we expected that KTs would detach from MT ends in these mutants. However, this was rare, and instead MT rescue at the KT happened in most cases (Maure et al., 2011). This raised the possibility that MT rescue at a KT may happen, following failure to achieve end-on attachment.

This possibility was also supported by the following observations. When end-on attachment was established, end-on pulling generally started at a full velocity (1.5–2.0 μm/min) immediately after the MT end had caught up with *CEN3* (Figures 1Ci and 1D). By contrast, when MT rescue happened at *CEN3*, the MT plus end paused at *CEN3* for an appreciable time (38 s on average; Figure 1D) prior to rescue, during which the MT showed neither shrinkage (exceeding 1.0 μm/min) nor growth (Figure 1Cii). This delay was not observed prior to the start of end-on pulling (Figure 1D) or MT rescue distal to the KT (Figures 1Ciii and 1D). Thus, there was a unique transition time that occurred prior only to MT rescue at the KT. Together, we hypothesized that end-on attachment may not always be successfully established such that it can support end-on pulling and, in response to its failure, MT rescue at the KT may take place. A KT may “sense” this failure during the phase of MT pausing and subsequently facilitate MT rescue at the KT to avoid loss of the KT-MT interaction.

### Microtubule Rescue Is Facilitated at a Kinetochore by Stu2, which Prevents a Loss of Kinetochore-Microtubule Attachment

We aimed to test the aforementioned hypothesis by preventing MT rescue at the KT. The following evidence suggested that Stu2 localizing at KT might promote MT rescue at the KT: (1) MT rescue distal to the KT was accompanied by transport of Stu2 from the KT to the MT plus end (Tanaka et al., 2005) and this process was indeed promoted by Stu2 (see the next section). Similarly, MT rescue at the KT may also be dependent on Stu2. (2) Stu2 and its orthologs could facilitate MT growth at the MT plus ends in vitro and in vivo (Al-Bassam et al., 2006; Brouhard et al., 2008; Ohkura et al., 2001). Other MT plus-end-tracking proteins (+TIPs), such as Bim1 and Bik1, might also play a similar role (Akhmanova and Steinmetz, 2008; Wolyniak et al., 2006) but, in contrast to Stu2, they did not show localization at *CEN3* that was associated with the lateral side of a spindle-pole MT (see Figure S1A available online).

However, we could not specifically prevent MT rescue at the KT using previously identified *stu2* mutants because, in such mutants, the number of spindle-pole MTs was also reduced, thus impairing KT-MT interactions from the beginning (Tanaka et al., 2005). Instead, we tried to partially reduce Stu2 function. For this, we coexpressed wild-type Stu2 and a mutant Stu2 lacking its first TOG domain (ΔTOG1) (Figure 2Ai); we consider this strain as a *stu2* hypomorphic mutant. The TOG domain of Stu2 binds tubulin and promotes MT polymerization (Al-Bassam et al., 2006). Stu2 is active only as a homodimer and the ΔTOG1 mutant can still form a dimer (Al-Bassam et al., 2006). We expected that, in a large subset of the homodimers, either one or both Stu2 molecules would lack the TOG1 domain in the hypomorphic mutant. As a control, we expressed two copies of wild-type Stu2.

Both the control and the *stu2* hypomorphic mutant cells frequently showed the initial *CEN3* interaction with the lateral side of a spindle-pole MT. Intriguingly, compared with the control, the *stu2* hypomorphic mutant showed less frequent MT rescue at *CEN3* (Figure 2Aii, category 3) and more frequent end-on pulling (Figure 2Aii, categories 1 and 2), suggesting that Stu2 indeed facilitates MT rescue at the KT (on *CEN3*). Consistent with this, in wild-type cells, the Stu2 intensity at KTs was reduced upon MT rescue at the KT (Figure S1B); thus, Stu2 seems to be transferred directly from the KT to the MT plus end. The *stu2* hypomorphic mutant also showed 16% of *CEN* detachment from the MT end (Figure 2Aiii), which was not found in the control (Figure 2Aii, category 4), supporting the hypothesis that MT rescue at the KT prevents loss of KT-MT attachment.

Moreover, in contrast to the control, the start of end-on pulling was delayed in some of the *stu2* hypomorphic cells (Figure 2Aii, category 2) with the MT pausing for 20–100 s (Figure 2Aiv and Figure S1C). Thus, the choice between MT rescue at the KT and establishment of end-on attachment might be dependent on the balance between the two mechanisms facilitating these options; when one mechanism fails, the other mechanisms may compensate, albeit after a short MT pausing.

If this notion is correct, we expect that KT detachment from the MT plus end would happen more frequently when both the mechanisms for MT rescue at the KT and establishment of end-on attachment are defective or inefficient. This was indeed the case as the double mutant of *stu2* hypomorph and the *ndc80* loop region showed KT detachment (similar to Figure 2Aiii) in 46% cases (Figure 2B, i and ii). Such a high rate of KT detachment was found not only in the engineered assay, but also in physiological conditions (Figure S1D). Collectively, our results suggest that end-on attachment often fails even in wild-type cells. In response to this failure, MT rescue happens at the KT, which is promoted by Stu2 at the KT and prevents loss of KT-MT attachment.

### Microtubule Rescue Distal to the Kinetochore Is Promoted by Stu2 Transport from the Kinetochore along the Microtubule Lateral Surface to Its Plus End

**Figure 3:**
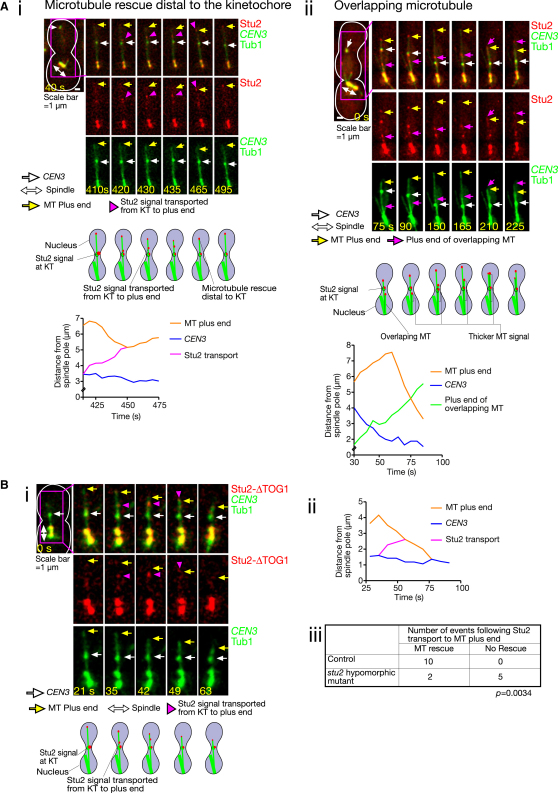
Microtubule Rescue Distal to the Kinetochore Is Promoted by Stu2 Transport from the Kinetochore along the Microtubule Lateral Surface to Its Plus End (A) MT rescue distal to the KT is distinct from the growth of an overlapping MT, and is preceded by Stu2 transport from the KT to the MT end. Time-lapse images of *P_MET3_*-*CDC20 P_GAL_-CEN3-tetOs TetR*-*GFP YFP*-*TUB1 STU2-3×CFP* (T4986) cells showing (i) Stu2 transport leading to MT rescue distal to the KT, and (ii) the growth of an overlapping MT. Schematic diagrams are shown at the bottom of the images. The graph shows MT length (orange), *CEN3*–spindle-pole distance (blue), and the position of Stu2 during its transport (i, magenta) and Stu2 at the end of an overlapping MT (ii, green). The cells were treated in the same way as in Figure 1C. Images were collected every 5 s using a common channel for GFP and YFP, and a separate channel for CFP. Zero time is set arbitrarily for the first panel. Scale bar, 1 μm. (B) Transport of a hypomorphic *stu2* mutant from the KT to the MT end often fails to promote MT rescue distal to the KT. (i) Time-lapse images, in which Stu2-ΔTOG1 transport did not lead to MT rescue. *P_MET3_*-*CDC20 P_GAL_-CEN3-tetOs TetR*-*3×CFP CFP*-*TUB1 STU2^+^ P_STU2_-stu2-ΔTOG1-3×GFP* (T9242) cells were treated as in Figure 1C, and images were collected every 7 s for CFP and GFP. Zero time is set arbitrarily for the first panel. Scale bar, 1 μm. (ii) The graph is plotted as in (Ai). (iii) The table shows the number of events in which Stu2 transport to the end of a shrinking MT did or did not lead to MT rescue distal to *CEN3*. See also Figure S2.

Is MT rescue distal to the KT also dependent on Stu2, similar to rescue that occurs at the KT? Indeed, as we reported previously, MT rescue distal to *CEN3* always happened coincidentally (24 out of 24 events) with arrival of Stu2 at the MT plus end after it had been transported from *CEN3* along the MT lateral surface (Tanaka et al., 2005) (Figure 3Ai). Stu2 transport along a MT was not a misinterpretation of the Stu2 signal at the end of an overlapping, growing MT, which could be discerned by a partly thicker MT signal (Figure 3Aii, 75–165 s) and a prior Stu2 movement from a spindle pole to *CEN3* (Figure 3Aii, 75–90 s). This conclusion was also confirmed by a separate experiment, using photobleaching of a MT region (Figure S2A). It appeared that Stu2 transport from a KT occurred and led to MT rescue distal to the KT not only in the engineered assay system (Figure 3Ai), but also in physiological conditions (Figure S2B). Stu2 transport from *CEN3* along a MT was observed with similar frequency during MT growth and shrinkage in the engineered assay (data not shown). If Stu2 arrived at the end of a growing MT, there was no obvious effect.

Given the above, Stu2 transport from the KT may indeed promote KT-dependent MT rescue distal to the KT, upon Stu2 arrival at the end of a shrinking MT. To address this, we investigated the *stu2* hypomorphic mutant cells (expressing both Stu2 wild-type and ΔTOG1 mutant; see Figure 2A), using the engineered assay. ΔTOG1, but not wild-type Stu2, was labeled with a green fluorescent protein (GFP) (Figure 3B). A control contained two copies of wild-type Stu2, one of which was labeled with GFP. In control cells the arrival of a GFP signal at the end of a shrinking MT after its transport from *CEN3* was always followed immediately by MT rescue (data not shown; similar to Figure 3Ai). In contrast, in the *stu2* hypomorphic mutant, the relevant MT often continued shrinking in spite of the arrival of a GFP signal at its end (Figure 3B). These results suggest that MT rescue distal to the KT is not only coincidental with, but also promoted by, Stu2 transport from the KT along the MT lateral surface.

### Kip3 Motor Activity Is Required for Stu2 Transport from the Kinetochore and for Microtubule Rescue Distal to the Kinetochore

**Figure 4:**
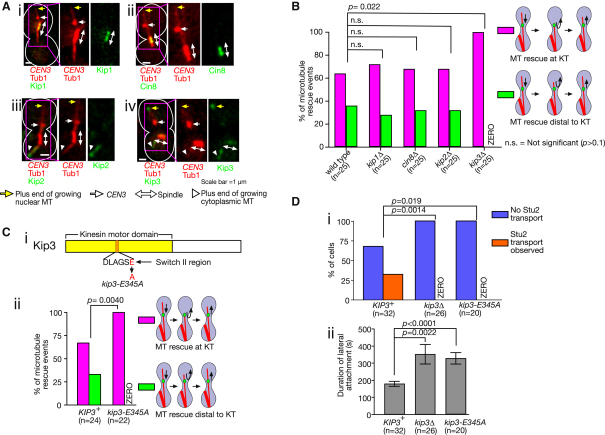
Kip3 Motor Activity Is Required for Stu2 Transport from the Kinetochore and for Microtubule Rescue Distal to the Kinetochore (A) Images of *KIP3-3×GFP* (i, T3981), *KIP2-3×GFP* (ii, T3926), *KIP1-3×GFP* (iii, T3822), and *CIN8-3×GFP* (iv, T4989) cells with *P_MET3_*-*CDC20 P_GAL_-CEN3-tetOs TetR*-*3×CFP CFP*-*TUB1*. The cells were treated in the same way as in Figure 1C. Images were collected using CFP and GFP channels. Scale bar, 1 μm. (B) Wild-type (T3531), *kip1Δ* (T2837), *cin8Δ* (T2838), *kip2Δ* (T2937), and *kip3Δ* (T2834) cells with *P_MET3_*-*CDC20 P_GAL_-CEN3-tetOs TetR*-*GFP YFP*-*TUB1*, showing proportion of rescue events taking place at the KT compared with rescue distal to the KT (left). The two kinds of MT rescue are depicted schematically (right). Cell preparation and image acquisition were carried out as in Figure 1C. (C) (i) The position of the mutation (*E345A*) in the switch II region of Kip3 is shown schematically. (ii) *KIP3*^+^ (T3531) and *kip3-E345A* (T4930) cells with *P_MET3_*-*CDC20 P_GAL_-CEN3-tetOs TetR*-*GFP YFP*-*TUB1* showing proportion of rescue events that took place at the KT compared with rescue distal to the KT. Cell preparation and image acquisition were carried out as in Figure 1C. (D) *KIP3*^+^ (T3680), *kip3*Δ (T3776), and *kip3-E345A* (T8657) cells with *P_MET3_*-*CDC20 P_GAL_-CEN3-tetOs TetR*-*3×CFP CFP*-*TUB1 STU2-3×GFP* showing the percentage of cells in which Stu2 was transported from the KT along a MT (i, orange), and the duration of *CEN3* interaction with the lateral side of a MT (ii, mean ± standard error of the mean). Cells were treated as in Figure 1C, and CFP and GFP images were acquired every 7 s for 20 min. See also Figure S3.

What factors promote Stu2 transport from the KT along a MT to facilitate MT rescue distal to the KT? Since Stu2 itself has no motor activity, a MT plus-end-directed motor might be involved in this process. The *S. cerevisiae* genome encodes four MT-dependent plus-end-directed motors, Kip1, Cin8, Kip2, and Kip3 (Hildebrandt and Hoyt, 2000). We investigated possible localization of these four motor proteins at *CEN3s* (associated with the lateral side of a MT), on the associated MTs, and on the spindle (Figure 4A). Kip1 and Cin8 localized on the spindle, but neither at *CEN3* nor on the *CEN3*-asssociated MT (Figure 4A, i and ii). Kip2 was detected on cytoplasmic MTs, but not within the nucleus (Figure 4Aiii), as reported previously (Tanaka et al., 2005). Meanwhile, the localization of Kip3 on the *CEN3*-asssociated MT was consistent with what was expected for a Stu2 transport facilitator (Figure 4Aiv); Kip3 was detected on the spindle and at the plus end of the *CEN3*-associated MT (and weakly along this MT; see Figure 6Aii), but not continuously at *CEN3*.

We next used yeast strains in which each candidate motor gene was deleted, and investigated the frequency of MT rescue at the KT and distal to the KT (Figure 4B). In *kip1*Δ, *cin8*Δ, and *kip2*Δ, the relative frequency of the two types of MT rescue was similar to a wild-type control. In contrast, in *kip3*Δ, MT rescue took place exclusively at the KT, suggesting that Kip3 may facilitate MT rescue distal to the KT.

If Kip3 facilitates MT rescue distal to the KT by promoting Stu2 transport along a MT, Kip3 motor activity may be required for this process. To test this, we introduced a mutation in the switch II region within the Kip3 motor domain (Figure 4Ci), which should impair ATP hydrolysis and the Kip3 motor activity (Browning et al., 2003). This mutant, *kip3-E345A*, was expressed at a similar level to wild-type *KIP3* (Figure S3A), and its function was indeed defective (Figures S3B and S3C). Crucially, in *kip3-E345A*, MT rescue occurred only at the KT (Figure 4Cii), suggesting that Kip3 motor activity is required to facilitate MT rescue distal to the KT.

We next addressed if Stu2 transport from the KT is dependent on Kip3. Neither *kip3*Δ nor *kip3-E345A* cells showed Stu2 transport from *CEN3* along a MT (Figure 4Di), despite these mutants having longer MTs (Figure S3C) on which *CEN3* was laterally associated for longer, giving greater potential for Stu2 transport (Figure 4Dii). Altogether, we conclude that the Kip3 motor activity is required for both Stu2 transport from the KT along a MT and subsequent MT rescue distal to the KT.

### Stu2 Is a Cargo Carried by a Fraction of Kip3 during Transport from a Kinetochore along a Microtubule

**Figure 5:**
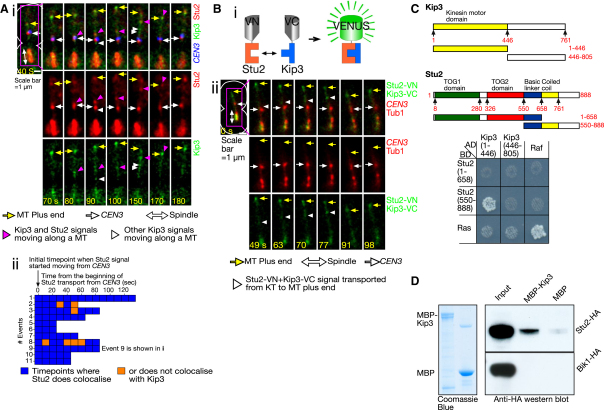
Stu2 Is Closely Associated with a Fraction of Kip3 during Its Transport from the Kinetochore along a Microtubule (A) *P_MET3_*-*CDC20 P_GAL_-CEN3-tetOs TetR*-*3×CFP STU2-4×mCherry KIP3-3×GFP* (T6038) cells were treated as in Figure 1C. Images were collected every 10 s using CFP, GFP, and mCherry channels. (i) Time-lapse images showing a representative example of colocalization of Stu2 and Kip3 during transport from the KT to the MT plus end. Scale bar, 1 μm. (ii) Graph showing 11 events of Stu2 transports during which Stu2 and Kip3 did (blue) or did not (orange) show colocalization. (B) (i) Schematic diagram showing the principle of the BiFC assay. (ii) Representative time-lapse images of a BiFC signal, generated by the close association between Stu2-VN and Kip3-VC, which moved along the MT toward its plus end. *P_MET3_*-*CDC20 P_GAL_-CEN3-tetOs TetR*-*3×CFP CFP*-*TUB1 STU2-VN KIP3-VC* cells (T6736) were treated as in Figure 1C. Images were collected every 7 s using CFP and YFP (for BiFC) channels. Scale bar, 1 μm. (C) The N-terminal half of Kip3 associates with the C-terminal half of Stu2 in a two-hybrid assay. The constructs, shown as diagrams (top), were fused with a DNA-binding domain (BD) or an activation domain (AD); amino acid residue numbers are in red. Equal numbers of cells, expressing the indicated combinations of constructs, were spotted on histidine drop-out plates and incubated for 48 hr (bottom). (D) Stu2 binds to Kip3 in vitro. MBP-Kip3 or MBP alone was immobilized on amylose beads. Coomassie blue lanes show the input proteins. After incubation with yeast extract from *STU2-HA* and *BIK1-HA* cells, HA-tagged proteins bound to the beads were detected by western blot. Input lanes were 1/250 of reaction. See also Figure S4.

Could Stu2 be a cargo that is transported by Kip3 along a MT? If so, we expect that Stu2 and Kip3 would move together from *CEN3* along a MT. Colocalization of Stu2 and Kip3 signals was transiently observed at *CEN3* (Figure 5Ai, 80 s). While Stu2 was transported from *CEN3* along a MT toward its plus end (90–170 s), Stu2 signals continued to colocalize with a fraction of Kip3 signals. During 11 events of Stu2 transport from *CEN3* toward the MT plus end, Stu2 signals colocalized continuously with Kip3 signals at most of the time points (20–130 s duration) (Figure 5Aii). In all 11 events of Stu2 transport, the relevant MTs showed rescue upon arrival of Stu2 at the MT plus end (e.g., Figure 5Ai, 170–180 s). While most Stu2 signals colocalized with a fraction of Kip3 signals during transport (Figure 5Ai, magenta arrowheads), other Kip3 signals showed movement along a MT without colocalization with Stu2 (white arrowheads).

Are Stu2 and Kip3 closely associated with each other during their transport? To test this, we used a bimolecular fluorescence complementation (BiFC) assay (Kerppola, 2008), in which Stu2 and Kip3 were fused with the N- and C-terminal halves of the YFP variant Venus fluorescent protein, respectively (Stu2-VN and Kip3-VC: Figure 5Bi and Figure S4A). If Stu2 and Kip3 associate closely, VN and VC might form a complete Venus protein, giving a fluorescent signal (Figure 5Bi). Pilot experiments confirmed that this indeed occurred rapidly once Stu2-VN and Kip3-VC associated closely (Figures S4B and S4C).

We investigated the interaction between Stu2-VN and Kip3-VC using the engineered assay (see Figure 1A). No Venus signals were detected at *CEN3* before it interacted with MTs extending from a spindle pole (data not shown). A Venus signal was often generated after *CEN3* was loaded on the lateral side of the MT, first weakly at *CEN3* (Figure 5Bii, 49 s) and then more clearly, immediately distal to *CEN3* (63–70 s). The Venus signal then moved toward the MT plus end (Figure 5Bii, 70–91 s), which led to rescue of the MT (91–98 s). We found five events of MT rescue distal to *CEN3*, all of which were accompanied by movements of Venus signals toward the MT ends. Such signals were not found in cells carrying either Stu2-VN or Kip3-VC alone (data not shown). We concluded that Stu2 and Kip3 are transported together, in close association, from *CEN3* to the MT plus end, leading to rescue of the MT.

The above result prompted us to investigate a possible physical interaction between Stu2 and Kip3. First, a yeast two-hybrid assay showed that the C terminus of Stu2 interacted with the N terminus of Kip3 (Figure 5C). Second, Stu2 (but not Bik1) in yeast cell extract showed a weak association with recombinant Kip3 in vitro (Figure 5D). This was not merely an indirect association mediated by MTs, because it also occurred after MTs were depolymerized with addition of nocodazole to both cell culture and the binding reaction (Figure S4D). Altogether, we suggest that a fraction of Kip3 molecules physically associate with Stu2 and transport it along a MT.

### Distinct Changes in the Levels of Stu2 and Kip3 Occur at the Microtubule Plus Ends, Facilitating Microtubule Rescue and Catastrophe, Respectively

**Figure 6:**
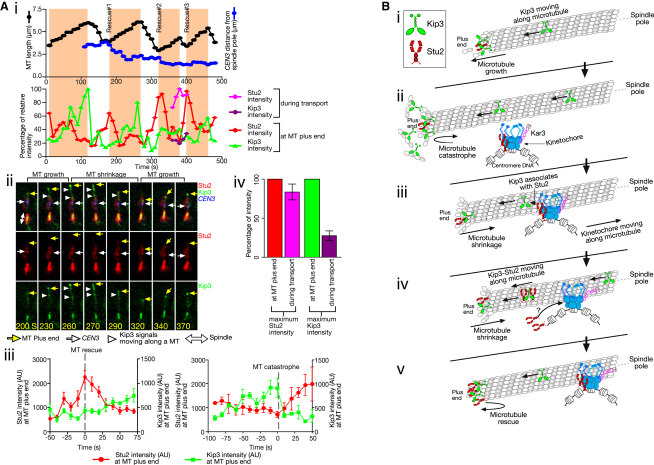
Distinct Changes in the Levels of Stu2 and Kip3 Occur at Microtubule Plus Ends, Facilitating Microtubule Rescue and Catastrophe, Respectively (A) *P_MET3_*-*CDC20 P_GAL_-CEN3-tetOs TetR*-*3×CFP STU2-4×mCherry KIP3-3×GFP* (T6038) cells were treated as in Figure 1C. Images were collected every 10 s using CFP, GFP, and mCherry channels. (i) The top graph shows the MT length (black) and the distance between *CEN3* and a spindle pole (blue) in a representative cell. Stu2 and Kip3 signals along the MT and at its plus end allowed an estimation of MT length. Phases of MT growth are shaded in orange. The lower graph shows the relative intensity (maximum = 100) of Stu2 (red) and Kip3 (green) at the MT plus end. The graph also compares the intensity of Stu2 (purple) with that of Kip3 (magenta) during cotransport of Stu2 and Kip3 along the MT. (ii) Time-lapse images (corresponding to 200–370 s in i) show Kip3 and Stu2 signals during growth and shrinkage of the relevant MT. Scale bar, 1 μm. (iii) Graphs show changes in the quantity of Stu2 and Kip3 (mean ± standard error of the mean) at the MT plus ends with respect to MT rescue and catastrophe (five and six events for left and right, respectively), which occurred at defined time zero. AU, arbitrary unit. (iv) The maximum intensities of Stu2 and Kip3 signals during their transport along the MT (mean ± standard error of the mean of 5 events for each), relative to their maximum intensity (set to 100) at the MT plus ends. (B) Summary of how Stu2 and Kip3 cooperate in KT-dependent MT rescue distal to the KT. (i) Kip3 frequently moves along a MT and accumulates at the MT plus end. (ii) As the MT becomes longer, more Kip3 accumulates at the MT plus end, causing MT catastrophe. (iii) After a KT interacts with the lateral side of the MT, Stu2 localizing at the KT binds a fraction of the Kip3 moving toward MT plus end. Meanwhile, the KT moves toward the MT minus end, driven by a kinesin-14 motor Kar3 (Tanaka et al., 2007). (iv) Stu2 and Kip3 are transported together toward the MT plus end. (v) The level of Stu2 increases at the MT end, causing MT rescue. See also Figure S5.

Stu2 and Kip3 are thought to facilitate microtubule rescue and catastrophe (conversion from growth to shrinkage), respectively, at the MT plus end (Al-Bassam et al., 2006; Brouhard et al., 2008; Gupta et al., 2006; Varga et al., 2006). How does the cotransport of Stu2 and Kip3 from *CEN3* change the levels of these proteins at the MT plus end, and how does this change regulate MT dynamics? Distinct changes in the levels of these two proteins were observed at the plus end of MTs during rescue and catastrophe (Figure 6A, i and ii). Stu2 signals showed maximum intensity upon MT rescue, while Kip3 signals showed peak intensity upon MT catastrophe (Figure 6Aiii). These distinct changes are consistent with the roles of Stu2 and Kip3 in MT rescue and catastrophe, respectively.

What mechanism leads to the distinct changes in the level of Kip3 and Stu2 at the MT plus end? Stu2 signals at the MT ends often increased rapidly prior to MT rescue; by contrast Kip3 signals increased more gradually during MT growth (Figure 6Aiii). The rapid increase of Stu2 signals took place either immediately after a single transport of Stu2 from *CEN3* toward the MT plus end (e.g., Figure 6Ai, Rescue #2 and #3) or after the plus end of a shrinking MT had caught up with *CEN3* (e.g., Figure 6Ai, Rescue #1), at which point Stu2 was presumably transferred directly from *CEN3* to the MT plus end (see Figure S1B). On the other hand, Kip3 moved along a MT more frequently than Stu2 (Figure 6Aii, white arrowheads), and only a fraction of Kip3 was associated with Stu2 as shown earlier (see Figure 5A). Our in vivo data support previous results in vitro (Varga et al., 2006, 2009) that Kip3 molecules move along a MT after being collected by the MT lateral surface (Figures S5A and S5B).

The unit amount of Stu2 moving along a MT was similar to the maximum amount found at the MT plus end, whereas the amount of Kip3 moving at any one time was much less than that usually present at the MT plus end (Figure 6Aiv). We estimated that a small number of Kip3 molecules (presumably one or two homodimers) could carry several Stu2 homodimers in a single transport event (Figure S5C). The results suggest that the level of Stu2 achieved following a single Stu2 transport event was sufficient for MT rescue distal to the KT (e.g., Figure 6Ai, Rescue #3, and Figure 6B). By contrast, to achieve the level of Kip3 at the MT plus end required for MT catastrophe, Kip3 must be supplied repeatedly because only a small number of Kip3 molecules move along a MT at any one time (Figures 6Aiv and 6B).

### Kinetochore-Dependent Microtubule Rescue and Subsequent Microtubule Extension Facilitate the Collection of Widely Scattered Kinetochores

MT rescue at the KT is crucial to prevent KT detachment from the MT plus end, as shown above. Apart from this role, does MT rescue at the KT provide any other benefits? Moreover, does MT rescue distal to the KT play any beneficial roles? To address these questions, we used a computer simulation to recapitulate the initial KT-MT interaction, observed in physiological conditions (Kitamura et al., 2007).

**Figure 7:**
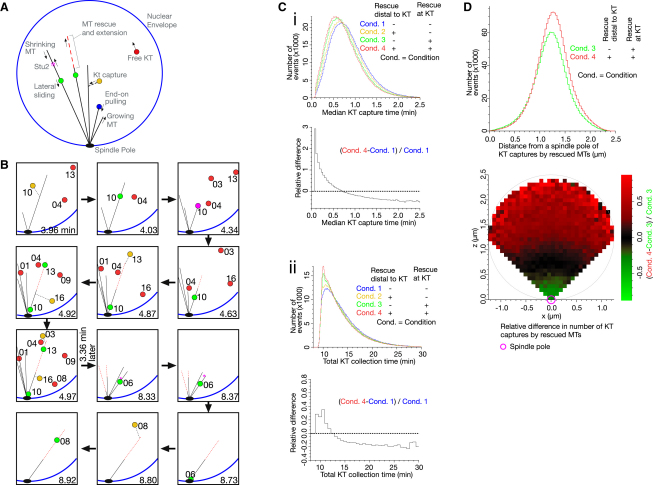
Computer Simulation of the Initial Kinetochore-Microtubule Interaction Reveals Benefits of Kinetochore-Dependent Microtubule Rescue (A) Diagram outlines a computer simulation that recapitulates the initial KT-MT interaction. KTs locate in the vicinity of a spindle pole before centromere DNA replication (not shown in this diagram). Upon centromere replication, KTs disassemble, and centromeres move away from a pole (Kitamura et al., 2007). KTs are then reassembled and interact with MTs extended from a spindle pole. Each KT undergoing different stages of KT-MT interaction is represented by a colored circle, as explained in the text. KTs are then transported to a spindle pole by MTs and subsequently tethered at the end of short MTs in the vicinity of the pole (not shown in this diagram). (B) Example of KT-dependent MT rescue leading to MT extension (red dashed line) and interaction with other KTs further away from a spindle pole. Time zero was defined as the time of replication of the first *CEN* (see Supplemental Experimental Procedures). The number, next to each KT, represents the number of *CEN*, on which the KT has been assembled. In this example, MT rescue happened at *CEN10* soon after 4.34 min, leading to interaction with *CEN13* and *CEN3*. MT rescue also occurred distal to *CEN6* soon after 8.37 min, leading to interaction with *CEN8*. The KT color code is shown in the text. A KT in magenta indicates MT pausing at the KT (see Figures 1Cii and 1D). For clearer presentation, one to three MTs, not associated with any *CEN*s, were omitted at time points 4.34–4.97 min. This example of simulation is also shown in Movie S1. (C) The effect of KT-dependent MT rescue on the overall state of KT collection by MTs. One million simulations were run with each combination of the presence and absence of MT rescue at the KT and distal to the KT (Conditions 1–4). Graphs in (i, top) and (ii, top) show the distribution of median KT capture time and total KT collection time (as defined in text; time zero as defined in B), respectively. Graphs in (i, bottom) and (ii, bottom) show relative difference between the presence and absence of KT-dependent MT rescue (rescue both at KT and distal to the KT), as defined by the indicated formulas. Bins along the x axis were 0.02 min (i, top), 0.04 min (i, bottom), 0.08 min (ii, top), and 0.64 min (ii, bottom). (D) MT rescue distal to the KT is particularly useful to collect other KTs that have drifted further away from a spindle pole. One million simulations were run in each condition. Graph (top) shows the distribution of the positions (distance from a spindle pole) for KT captures by rescued MTs (along the extended MT region following rescue), in Condition 3 (rescue only at the KT) and Condition 4 (rescue both at the KT and distal to the KT). The binning along the x axis was 0.03 μm. The heat map (bottom) shows the spatial distribution in the nucleus of relative difference in the number of KT captures by rescued MTs (as defined above) between Conditions 3 and 4 as defined by the formula. The circle (gray) represents the nuclear envelope. KT capture events along the y axis were evaluated to project the relative difference to the x–z plane. See also Figure S6.

KTs localize in the vicinity of a spindle pole in the G1 phase, but upon centromere DNA replication they disassemble and centromeres move away from the pole (Kitamura et al., 2007). In our simulation, we assumed that this happened to 16 centromeres of *S. cerevisiae* over 7.5 min, following their defined replication timing (Yabuki et al., 2002). One minute after replication of each centromere, the KT was reassembled (Figure 7A, red dot) and subsequently caught on the lateral surface of a MT extending from a spindle pole (green dot) (Kitamura et al., 2007); this capture process was facilitated by short MTs transiently generated at the KT (dashed line from a yellow dot) (Kitamura et al., 2010). KTs were then transported toward a spindle pole by sliding along a MT (Figure 7A, green dot) and, subsequently, by end-on pulling (blue dot) once end-on attachment was established (Tanaka et al., 2007). Most parameter values for MT dynamics and centromere motions were defined in our previous studies of live-cell imaging and electron tomography (Kitamura et al., 2007, 2010; Tanaka et al., 2005, 2007).

In “wild-type” conditions, MT rescue happened both at the KT and distal to the KT, 7.1 ± 2.6 times (mean ± standard deviation) in individual cells (Figure S6A). The simulation was also run with either or both types of MT rescue “switched off.” In “wild-type” conditions, we found that regrowth and extension of MTs due to both types of KT-dependent MT rescue often led to MT interactions with other KTs further away from a spindle pole (Figure 7B, Figure S6A, and Movie S1). Accordingly, KT-dependent MT rescue effectively shortened: (1) the median KT capture time (measured in individual cells), i.e., the time from reassembly of each KT until its interaction with a spindle-pole MT (Figure 7Ci and Figure S6B); and (2) total KT collection time, i.e., the time required for collection of all KTs to the vicinity of a spindle pole by spindle-pole MTs (Figure 7Cii and Figure S6B; time zero, replication of the first *CEN*, which caused its detachment from a spindle pole). MT rescues both at, and distal to, the KT contributed to shortening these parameters.

In what kind of cell population are such effects greatest? KT-dependent MT growth greatly reduced (by up to 50%) the median KT capture time in cell populations where it was longest, i.e., more than 1.5 min (Figure 7Ci, bottom). This was also the case for the cell population with longest total KT collection time, i.e., more than 20 min, reduced by up to 20% (Figure 7Cii, bottom). Thus, KT-dependent MT rescue contributed proportionally more to the reduction of KT collection time when KT collection by spindle-pole MTs was particularly slow. Hence, KT-dependent MT rescue provides a fail-safe mechanism to diminish a long delay in KT collection by spindle-pole MTs.

Note that most KT captures by rescued and extended MTs occurred during the period of 2.5–11 min of a simulation (data not shown). For this type of capture to occur, another KT (other KTs) must have been in the process of transport toward a spindle pole on its associated MTs in order to cause MT rescue. On the other hand, when there was a delay, e.g., >20 min, in collecting all KTs by MTs, usually only one KT remained uncaught by MTs, and KT captures by rescued and extended MTs were rare during this late period (data not shown). We reason that earlier KT capture by rescued MTs can reduce the probability that any KT subsequently remains uncaught for a long time, e.g., beyond 20 min.

We next addressed whether MT rescue distal to the KT played any unique role in ensuring KT-MT interaction. Such a unique role was revealed when we analyzed the position of KTs upon their initial encounter with the extended regions of rescued spindle-pole MTs, in the presence and absence of MT rescue distal to the KT (rescue at the KT occurred in both these conditions) (Figure 7D). In its presence (Condition 4), a larger number of KTs were collected by rescued MTs (Figure 7D, top). Furthermore, when KTs localized further away from a spindle pole, KT collection by rescued MTs occurred proportionally more frequently (by up to 80%) in the presence of MT rescue distal to the KT (Figure 7D, bottom; also refer to Figure S6C). Thus, MT rescue distal to the KT is particularly useful for the collection of KTs that have drifted further away from a spindle pole.

## Discussion

In early mitosis, a KT initially interacts with the lateral surface of a spindle-pole MT (Rieder and Alexander, 1990; Tanaka et al., 2005). During this lateral attachment, the KT promotes rescue of its associated MT (Tanaka et al., 2005). Using budding yeast as a model organism, we have studied mechanisms of KT-dependent MT rescue as well as its benefits for KT-MT interactions. In our study, we first used live-cell imaging in the engineered assay (Figures 1–6), confirmed our conclusions in physiological conditions (Figures S1D and S2B), and further developed our study using computer simulations (Figure 7).

### Cooperative Action of Opposing Regulators

KT-dependent MT rescue occurs either before or after the plus end of a shrinking MT reaches the KT. Stu2 promotes both these types of KT-dependent MT rescue. For rescue distal to the KT, Stu2 is transported from the KT along a MT by Kip3 and triggers MT rescue at the MT plus end. On the other hand, rescue at the KT does not require Kip3; perhaps Stu2 is transferred directly from the KT to the MT end to facilitate MT rescue. Consistent with the role of Stu2 in MT rescue, XMAP215 (Stu2 ortholog in *Xenopus*) promotes MT polymerization in vitro (Brouhard et al., 2008); Stu2 orthologs in *Drosophila* and *Arabidopsis* facilitate MT dynamics at MT plus ends in vivo (Brittle and Ohkura, 2005; Kawamura and Wasteneys, 2008); and Stu2 directly binds tubulin (Al-Bassam et al., 2006).

Promoting KT-dependent MT rescue is one of multiple roles of Stu2 localizing at the KT. Stu2 also plays a central role in promoting MT generation at the KT before the KT interacts with a spindle-pole MT (Kitamura et al., 2010). The KT-derived MTs subsequently interact with spindle-pole MTs along their length, facilitating KT loading on the lateral side of spindle-pole MTs. Shortly after this, some of Stu2 at the KT appear to diffuse away quickly in both directions along spindle-pole MTs (Kitamura et al., 2010). This Stu2 motion is clearly distinct from the Stu2 transport that occurs only toward the MT plus end.

Meanwhile, Kip3 molecules bind the MT lateral surface and, once bound, they move along a MT using their activity of a MT plus-end-directed motor (Gupta et al., 2006; Varga et al., 2006). Subsequently, Kip3 accumulates at the MT plus ends and causes MT catastrophe and depolymerization (Gupta et al., 2006; Varga et al., 2006). Kip3-dependent MT depolymerization happens more frequently on longer MTs in vitro (Varga et al., 2006, 2009); we showed that Kip3 indeed enhances MT catastrophe in a MT length-dependent manner in vivo (Figures S5A and S5B).

Our results suggest that Kip3 drives Stu2 transport from the KT along a MT; thus, they act cooperatively in vivo to promote MT rescue distal to the KT. How does Kip3 reach the KT before collecting Stu2 at the KT and subsequently transporting it along a MT toward the plus end? We think that, when Kip3 molecules move along a MT and pass by *CEN3*, they collect Stu2 from *CEN3* (see Figure 6Biii); in some examples we indeed observed that a Kip3 signal moved toward *CEN3* along a MT, collected Stu2 at *CEN3*, and further moved toward the MT plus end together with Stu2 (data not shown). However, when Kip3 first appeared around *CEN3* (e.g., Figure 5Ai, 80 s), we could not determine whether Kip3 was first loaded on the KT (at *CEN3*) or on the MT in the vicinity of *CEN3* before starting a Stu2 transport along the MT.

In contrast to their cooperative action for Stu2 transport, Kip3 and Stu2 on their own likely play opposing roles in MT dynamics at the MT plus end (Severin et al., 2001), as discussed above. How is their cooperative action linked to their opposing actions at the MT plus ends? When the KT associates with the lateral side of a MT, a small fraction of the Kip3 motors picks up Stu2 molecules from the KT and carries them along the MT to its plus end. In a single transport event, presumably several Stu2 homodimers are transported by one or two Kip3 homodimers. There is sufficient Stu2 in a single transport event to cause MT rescue, whereas multiple Kip3 movements, plus its accumulation at the MT end, are necessary for MT catastrophe. Because of this difference, Stu2 and Kip3 can promote MT rescue and catastrophe, respectively, with distinct timing. It is unclear how Kip3 can carry several Stu2 molecules at one time; Kip3 may have multiple Stu2 association sites or Stu2 homodimers may be able to associate with each other during transport.

### How to Back Up a Process that Fails Even in Normal Conditions

Lateral KT-MT attachment must be eventually converted to the end-on attachment, which is presumably necessary for sister KT biorientation (Maure et al., 2011; Tanaka, 2010). However, KT-dependent MT rescue extends the duration of lateral attachment. Why then do cells need to undergo such MT rescue? A MT rescue at the KT seems to be a response to failure of establishment of end-on attachment. We reason that the conversion from lateral to end-on attachment is an elaborate process requiring a drastic change in the KT-MT interface (Maure et al., 2011; Tanaka, 2010); this process therefore often fails, even in wild-type cells. It seems that, as backup measures, Stu2 at the KT promotes MT rescue so that at least loss of KT-MT attachment is avoided.

Prior to MT rescue at the KT, the MT plus end shows a short pause at the KT. During this period, the KT may “sense” a failure in establishing end-on attachment that could support end-on pulling. Presumably, the dynamic nature of MTs precludes an extended pausing; therefore, MT rescue is the only way to enable another chance of establishing end-on attachment (after subsequent MT growth, catastrophe, and shrinkage), without losing the initial KT-MT interaction.

### “Altruistic” Actions of Kinetochores

Preventing loss of KT-MT attachment is not the only benefit of KT-dependent MT rescue. Our computer simulation suggests that regrowth and extension of MTs, due to KT-dependent MT rescue, often help them to interact with other KTs further away from a spindle pole. KTs are then transported to a spindle pole by their associated MTs. Thus, KTs show “altruistic” actions in collecting other KTs by promoting MT rescue. This action reduces the time required for collection of a whole set of KTs to the vicinity of a spindle pole.

Intriguingly, this reduction was greatest in cells where this time period was longer. Therefore KT-dependent MT rescue provides a useful fail-safe mechanism, i.e., it reduces the population of cells that shows a long delay in the collection of all KTs by spindle-pole MTs. Although MT rescues, both at and distal to the KT, contribute to this effect, rescue distal to the KT plays proportionally more important roles in collecting KTs that have drifted further away from a spindle pole, thus reinforcing the abovementioned fail-safe mechanism.

Furthermore, both in vitro and in vivo results suggest that Kip3 density is higher along the distal region of MTs (i.e., further away from a spindle pole; Figures S5A and S5B) (Varga et al., 2006, 2009). This property of Kip3 could lead to more frequent Stu2 transport events from KTs when the KTs are associated with more distal regions of MTs. By this mechanism, MT rescue distal to the KT may help to maintain particularly long MTs, which could effectively collect widely scattered KTs in the nucleus.

Why is it important to reduce the long delay in KT collection to the vicinity of a spindle pole? Is there a time limit for the KT collection? We presume that it is important to collect all KTs, prior to spindle-pole separation that occurs at 30–40 min (data not shown; time zero, replication of the first *CEN*). Once spindle poles separate, many MTs from opposite poles interact with each other and would become less capable of searching for free KTs in various directions.

### Concluding Remarks

Is KT-dependent MT rescue in early mitosis (prometaphase) also found in other organisms? This issue has not yet been explored extensively. Nonetheless, Msps and Dis1/Alp14, which are Stu2 orthologs in *Drosophila* and fission yeast, respectively, also localize at the KT in early mitosis (Buster et al., 2007; Garcia et al., 2001; Hsu and Toda, 2011; Nakaseko et al., 2001). Thus, they may also facilitate KT-dependent MT rescue while KTs initially localize on the lateral surface of spindle-pole MTs. Meanwhile, transport of +TIPs by motor proteins, analogous to Stu2 transport by Kip3, is found in other systems and organisms. Indeed, CLIP-170-like +TIP proteins Bik1 and Tip1 are transported by Kip2 and Tea2 motors along cytoplasmic MTs in budding and fission yeast, respectively (Busch et al., 2004; Carvalho et al., 2004).

In conclusion, our studies reveal intriguing mechanisms and benefits of KT-dependent MT rescue. The dynamics of KT-associated MTs are regulated by both cooperative and opposing actions between Kip3 and Stu2. These actions are coordinated in a sophisticated manner to accomplish their functions at the correct time and place. KT-dependent MT rescue provides a number of beneficial fail-safe mechanisms, including preventing a loss of KT-MT interaction upon failure of conversion from lateral to end-on KT-MT attachment. Furthermore, KT-dependent MT rescue facilitates collection of KTs further afield, thus diminishing a long delay in collecting a whole set of KTs to the vicinity of a spindle pole.

## Experimental Procedures

### Yeast Strains and Cell Culture

The background of yeast strains (W303) and methods for yeast culture were as described previously (Tanaka et al., 2007). Unless otherwise stated, cells were cultured at 25°C in YP medium containing glucose, and yeast genes were tagged at their C termini at their original gene loci by a one-step PCR method using 3× GFP (pSM1023), 4× mCherry (pT909), and 3× CFP (pT769) cassettes as PCR templates. See more details in Supplemental Experimental Procedures.

### Live-Cell Imaging

The procedures for time-lapse fluorescence microscopy were described previously (Kitamura et al., 2007; Tanaka et al., 2007). Time-lapse images were collected at 25°C (ambient temperature). For image acquisition, we used a DeltaVision RT microscope (Applied Precision), UPlanSApo 100× objective lens (Olympus; NA 1.40), a CoolSNAP HQ CCD camera (Photometrics), and softWoRx software (Applied Precision). We acquired five to nine (0.7 μm apart) z sections, which were subsequently deconvoluted and analyzed with softWoRx and Volocity (Improvision) software. For figures, Z stacks were projected to two-dimensional images. To evaluate the length of MTs and position of centromeres, we took account of the distance along the z axis as well as distance on each z plane. See more details in Supplemental Experimental Procedures.

### Computer Simulation

A computer model of the initial KT-MT interaction was created, based on configuration in the physiological conditions (Kitamura et al., 2007). The model was computed as a discrete simulation of a series of events, and all objects (MTs, KTs, and Stu2) were located in a three-dimensional space. Each MT was a line segment extending into the nucleus from the spindle pole, and each KT was a point inside the nucleus. The interaction between KT-generated and spindle-pole MTs was simplified by assuming a certain capture radius, *R*_KT_ (0.4 μm), around each KT. If a KT was found at a distance *R*_KT_ from any part of a spindle-pole MT, a KT-derived MT connected the KT to this spindle-pole MT by the shortest distance and brought the KT toward the spindle-pole MT, usually on its lateral side. Time zero was defined as the time of replication of the first *CEN* (*CEN2*) (Yabuki et al., 2002) and therefore its detachment from a spindle pole (Kitamura et al., 2007). The code for the simulation was written in Perl and simulations were run in a Linux environment. We ran 1 million individual simulations in each condition. See more details in Supplemental Experimental Procedures.

## Supplemental Information

Document S1. Six Figures and Supplemental Experimental Procedures

Movie S1. An Example of Simulation in “Wild-Type” Conditions, i.e. in the Presence of MT Rescue Both at the KT and Distal to the KT (Condition 4 in Figures 7C and 7D) The same example is also presented in Figure 7B. Projections for x-z and x-y planes are shown at the left and right, respectively. Once each *CEN* experiences detachment from a spindle pole (yellow dot), reattach to a MT and comes back at a spindle pole, a corresponding number at top becomes blue.
