# Supplementary material for: Kinetochore-Dependent Microtubule Rescue Ensures Their Efficient and Sustained Interactions in Early Mitosis
Source: Dev Cell. 2011 Nov 15;21(5-5):920–33. doi: 10.1016/j.devcel.2011.09.006 (PMC3277888; doi:10.1016/j.devcel.2011.09.006)
Supplement: Document S1. Six Figures and Supplemental Experimental Procedures [file mmc1.pdf]

Developmental Cell, Volume *21*

Supplemental Information

Kinetochore-Dependent Microtubule Rescue

Ensures Their Efficient and Sustained

Interactions in Early Mitosis

Sapan R. Gandhi, Marek Gierliński, Akihisa Mino, Kozo Tanaka, Etsushi Kitamura, Lesley Clayton, and Tomoyuki U. Tanaka

Inventory of Supplemental Information

Figure S1 (related to Figure 2)
(A) Stu2, but not Bik1 or Bim1, localizes at *CEN3* that is associated with the lateral surface of a spindle-pole MT.
(B) The amount of Stu2 at the KT decreases upon MT rescue distal to, and at KT.
(C) End-on attachment/pulling is preceded by MT pausing in some *stu2* hypomorphic mutant cells.
(D) The double mutant *stu2* hypomorph plus the *ndc80* loop deletion shows frequent KT re-detachment from MTs in physiological conditions.

Figure S2 (related to Figure 3)
(A) A photo-bleaching experiment demonstrates that MT rescue distal to the KT is distinct from growth of an overlapping spindle-pole MT and a possible KT-derived MT.
(B) Evidence that Stu2 transport from the KT to the MT end leads to MT rescue in physiological conditions.

Figure S3 (related to Figure 4)
(A) Western blot analysis of *kip3-E345A* and wild-type *KIP3* expression.
(B) *kip3-E345A* is synthetically lethal with a *kar3-64* mutant.
(C) *kip3*Δ and *kip3-E345A* show longer MTs than wild-type *KIP3*.

Figure S4 (related to Figure 5)
(A) Western blot analysis of *KAR3-VC*, *KIP3-VC* and *STU2-VN* expression.
(B) Venus could be formed rapidly to emit its signal when Stu2-VN and Kip3-VC first became closely associated.
(C) *STU2-VN KIP3-VC*, but not *STU2-VN KAR3-VC*, gives BiFC signals at the MT end.
(D) Stu2 binds Kip3 *in vitro* in the presence of nocodazole.

Figure S5 (related to Figure 6)
(A) A larger amount of Kip3 is present at the ends of longer MTs.
(B) Longer MTs show a higher frequency of MT catastrophe and this correlation is partly dependent on Kip3.
(C) Estimating the number of Stu2 and Kip3 molecules in a single transport.

Figure S6 (related to Figure 7)
(A) Various evaluations of KT-MT interactions from simulations.

(B) The effects of KT-dependent MT rescue on overall KT collection are mainly due to capture of other KTs by the extended MT region following MT rescue.
(C) MT rescue distal to the KT is particularly useful for collecting KTs that have drifted further away from a spindle pole.

Movie 1 (related to Figure 7)

An example of simulation in the presence of MT rescue both at the KT and distal to the KT.

Supplemental Experimental Procedures

Supplemental References

Supplemental Figures

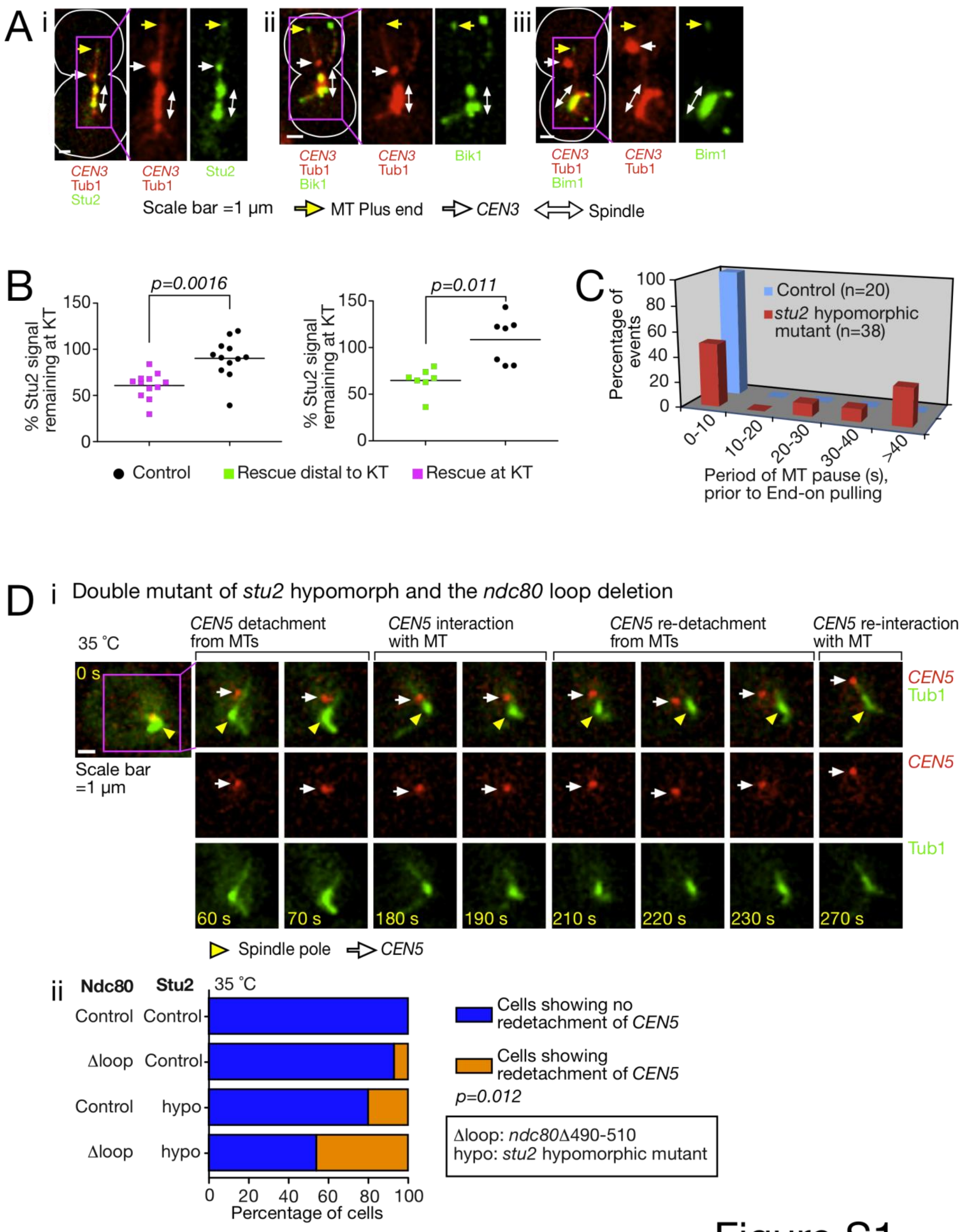

Figure S1

Figure S1 (associated with Figure 2)

(A) Stu2, but not Bik1 or Bim1, localizes at *CEN3* that is associated with the lateral surface of a spindle-pole MT.

STU2-3×GFP (T3680, i), *BIK1-3×GFP* (T3674, ii) *and BIM1-3×GFP* (T3766, iii) cells with P_{MET3}*-CDC20* P_{GAL}*-CEN3-tetOs TetR-3×CFP CFP-TUB1* were treated as in Fig 1C. GFP and CFP images were collected. Image panels show cells with *CEN3* associated with the lateral surface of a spindle-pole MT.

(B) The amount of Stu2 at the KT decreases upon MT rescue distal to, and at the KT.

P_{MET3}*-CDC20* P_{GAL}*-CEN3-tetOs TetR-GFP YFP-TUB1 STU2-3×CFP* (T4986) cells were treated and time-lapse images were acquired as in Fig 3A. See examples of time-lapse images in Fig 3A. Graphs show changes in intensity of the Stu2 signal on *CEN3* after MT rescue distal to the KT (on *CEN3*; green, left) and at the KT (magenta, right). The amount of change was shown relative to the intensity of the Stu2 signal on *CEN3* prior to rescue. For rescue at the KT, the Stu2 signal was quantified just before the MT end caught up with *CEN3* and just after the MT end had visibly started growing away from *CEN3*. For rescue distal to the KT, the Stu2 signal was quantified before and after a Stu2 signal had begun transport and moved away from *CEN3*. For controls, the change in Stu2 signal was similarly quantified at corresponding time points in the same microscopy field, but in the cells where *CEN3* was on a MT lateral surface and MT rescue/Stu2 transport were not observed during the relevant time windows.

(C) End-on attachment/pulling is preceded by MT pausing in some *stu2* hypomorphic mutant cells.

T9323 and T9345 (see genotypes in Fig 2A legend) were treated and analyzed as in Fig 2A. Graph showing the period of MT pausing (as defined in Fig 1D legend), prior to end-on attachment/pulling in the *stu2* hypomorphic mutant and control cells.

(D) The double mutant *stu2* hypomorph plus the *ndc80* loop deletion shows frequent KT re-detachment from MTs in physiological conditions.

$NDC80^{+}$ P_{STU2}-$STU2^{+}$ (T9692), *ndc80Δ490-510* P_{STU2}-$STU2^{+}$ (T9634), $NDC80^{+}$ P_{STU2}*-stu2ΔTOG1* (T9621) and *ndc80Δ490-510* P_{STU2}*-stu2ΔTOG1* (T9555) cells with *CEN5-tetOs TetR-3×CFP Venus-TUB1* $STU2^{+}$ were treated with α factor and released to fresh YPD medium at 35°C. Images were taken 30 minutes after cells were released to YPD medium at 35°C. CFP and Venus images were acquired every 10 sec at 35°C. (i) Representative time-lapse images of a T9555 cell. (ii) Graph shows the proportion of cells showing *CEN5* re-detachment from spindle pole MTs, following their initial interaction (orange bars). Re-detachment was scored if a cell showed *CEN5* re-detachment from MTs (following the initial KT-MT interaction) before *CEN5* was transported to the vicinity of a spindle pole (<0.5 μm) or soon after (<50 sec) its arrival in the vicinity. Note that Ndc80 control= $NDC80^{+}$ wild type, Stu2 control=two copies of wild-type *STU2* (one is at an auxotroph locus), Ndc80 Δloop=*ndc80Δ490-510* and Stu2 hypo (hypomorphic mutant)= P_{STU2}*-stu2ΔTOG1* (at an auxotroph locus) plus wild-type $STU2^{+}$. The number of cells analyzed: n= 12, 15, 15 and 13 (top to bottom).

Results: We addressed if the double mutant, *stu2* hypomorph plus the *ndc80* loop deletion, showed defects in KT-MT interactions in physiological conditions, similar to those in the engineered assay (see Fig 2B). In a control, single mutants (*stu2* hypomorph or the *ndc80* loop deletion) and the double mutants, *CEN5* detached from MTs and moved away from a spindle pole in a similar timing (data not shown), presumably upon *CEN5* DNA replication (Kitamura et al., 2007). Approximately in 2-3

min, *CEN5* interacted again with MTs in all these cells (except for about 10 % of the double mutant cells that showed a further delay in this interaction). However, in the double mutant, *CEN5* often showed re-detachment from MTs (i). Such re-detachment was less frequent in single mutants and not observed in control cells (ii). In the double mutant and single mutants, re-detached *CEN5* showed interaction with spindle-pole MTs again in 1-2 min (e.g. 270 sec in i).

Discussion: We also addressed if MT dynamics were altered in mutants of *stu2* hypomorph and the *ndc80* loop deletion. We measured nucleation frequency of cytoplasmic MTs (extending toward the cell cortex and showing maximum length > 2 μm) in the three (double and singles) mutants using physiological conditions. These mutants showed similar nucleation frequency to control cells (data not shown). We also measured other parameters of MT dynamics, such as the polymerization speed and maximum length of nuclear MTs, using the engineered assay (see Fig 1A). These parameters were also similar in the mutant and control cells (data not shown). Based on these results, we reason that KT-dependent MT rescue is more sensitive to a reduced Stu2 function than are other MT dynamics studied here.

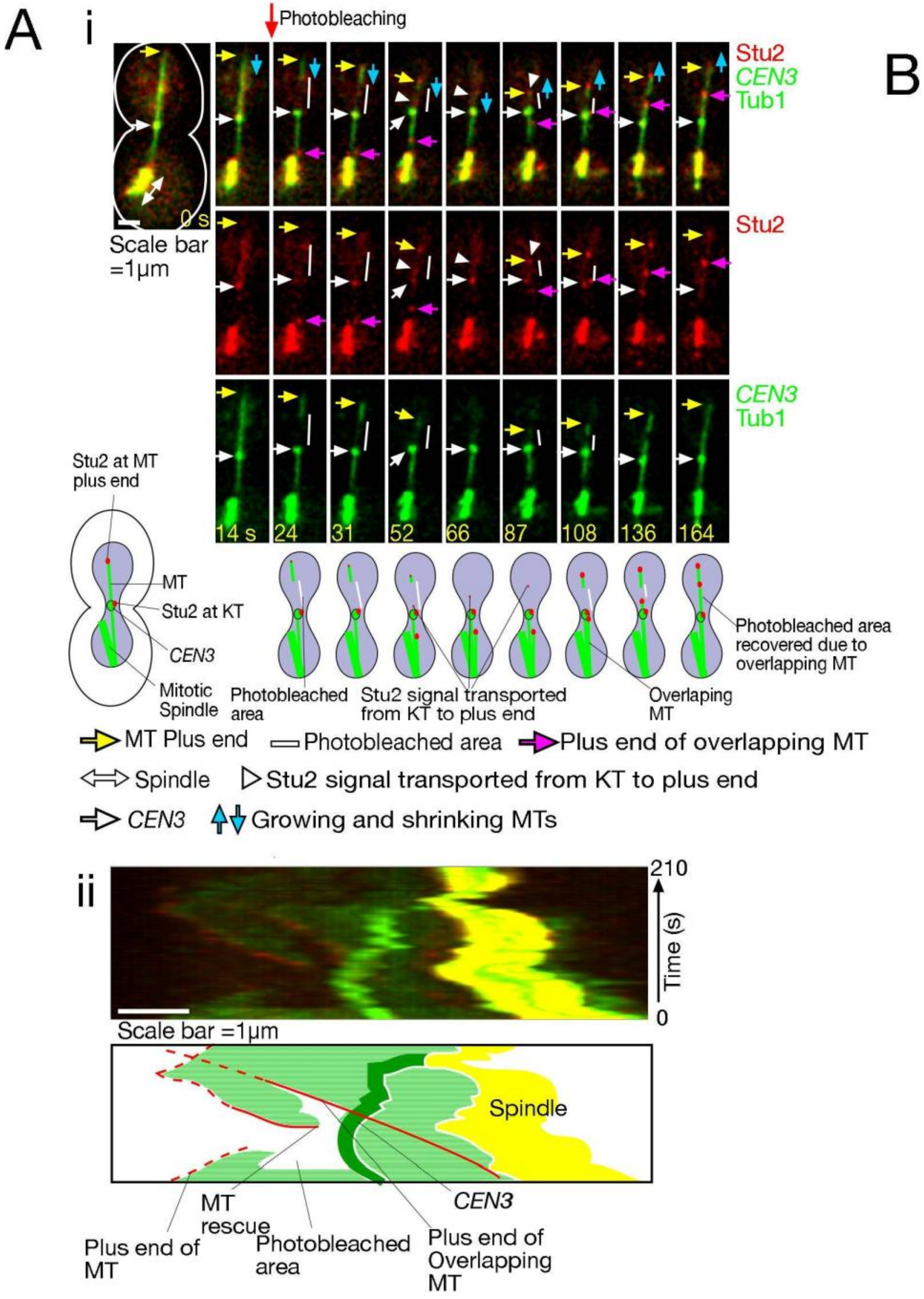
A
i
Photobleaching
Stu2
CEN3
Tub1
0 s
Scale bar =1μm
14 s
24
31
52
66
87
108
136
164
Stu2 at MT plus end
MT
Stu2 at KT
CEN3
Mitotic Spindle
Photobleached area
Stu2 signal transported from KT to plus end
Overlapping MT
Photobleached area recovered due to overlapping MT
MT Plus end
Photobleached area
Plus end of overlapping MT
Spindle
Stu2 signal transported from KT to plus end
CEN3
Growing and shrinking MTs
ii
210
Time (s)
0
Scale bar =1μm
Spindle
MT rescue
Plus end of MT
Photobleached area
CEN3
Plus end of Overlapping MT

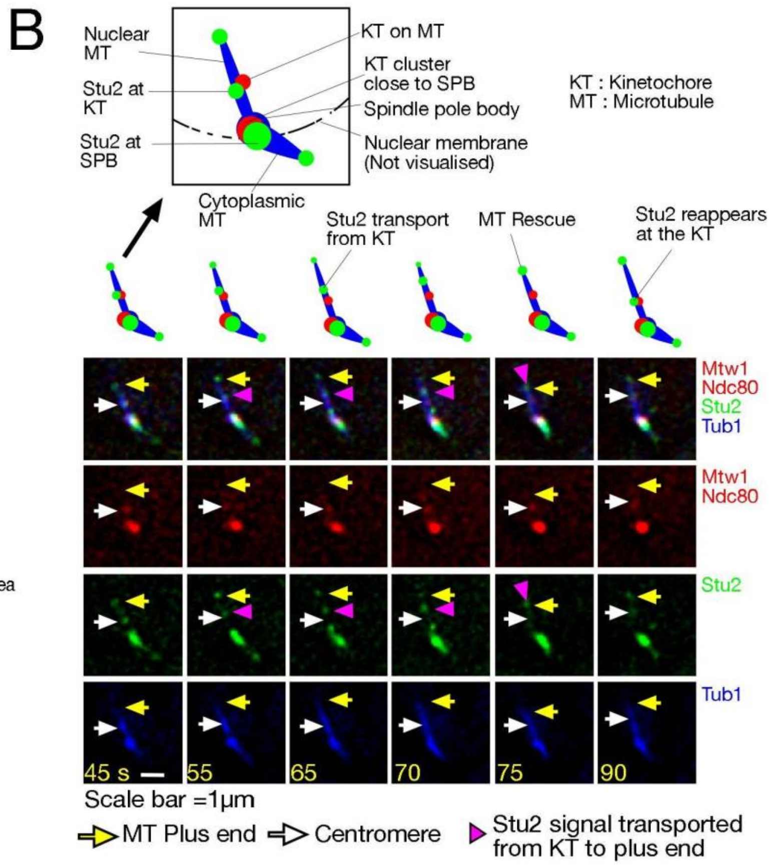
B
Nuclear MT
Stu2 at KT
Stu2 at SPB
KT on MT
KT cluster close to SPB
Spindle pole body
Nuclear membrane (Not visualised)
Cytoplasmic MT
KT : Kinetochore
MT : Microtubule
Stu2 transport from KT
MT Rescue
Stu2 reappears at the KT
Mtw1
Ndc80
Stu2
Tub1
45 s
55
65
70
75
90
Scale bar =1μm
MT Plus end
Centromere
Stu2 signal transported from KT to plus end

Figure S2

Figure S2 (associated with Figure 3)

(A) A photo-bleaching experiment demonstrates that MT rescue distal to the KT is distinct from growth of an overlapping spindle-pole MT and a possible KT-derived MT.

Time-lapse images (i) and kymograph (ii) of a T4986 cell (see Fig 3A legend) showing an example of MT rescue distal to the KT. Schematic diagrams are shown at bottom of the images (i). The cells were treated in the same way as Fig 1C. Images were collected as in Fig 1C, but every 7 sec after photo-bleaching.

Results: To obtain further evidence that rescue of a *CEN3*-associated MT is indeed distinct from growth of an overlapping MT from a spindle pole, we photo-bleached a small MT region between *CEN3* and the MT plus end (between 14 and 24 sec). The photo-bleached area of the MT was not filled when the relevant MT showed 'rescue' (87-108 sec time points). In contrast, the photo-bleached gap was filled when subsequently an overlapping MT grew out (108-164 sec time points). These two events were also clearly distinct when displayed as a kymograph. Thus, rescue of a *CEN3*-associated MT (first event) and growth of an overlapping MT (second event) are discernibly different.

Discussion: We previously reported that MTs can be generated at *CEN3* with distal plus ends before *CEN3* becomes associated with a MT extending from a spindle pole (Kitamura et al., 2010). Could the 'rescue' of a *CEN3*-associated MT be a misinterpretation of a MT growth from *CEN3*? This was not the case because 1) the latter would fill the gap of a photo-bleached area (in this Figure) and 2) MTs are no longer generated at *CEN3* once it becomes associated with a MT extending from a spindle pole (Kitamura et al., 2010).

(B) Evidence that Stu2 transport from the KT to the MT end leads to MT rescue in physiological conditions.

Time-lapse images of *CFP-TUB1 NDC80-4×mCherry MTW1-4×mCherry STU2-3×GFP* (T8471) showing transport of Stu2 from a KT, along a MT towards its plus end, leading to rescue of the MT. The cells were treated with α-factor in YP medium containing glucose for 2.5 hrs and released into fresh YP medium containing glucose. In 30 minutes after release, images were acquired every 5 sec, using CFP (tubulin), GFP (Stu2) and mCherry (Mtw1, Ndc80; KT components) channels. Scale bar, 1 μm. The time-lapse images are interpreted using the schematic diagram above.

Results: We investigated the behaviour of Stu2 in physiological conditions without any artificial centromere regulation (i.e. not using the engineered assay) or cell cycle arrest. After KTs were reassembled on centromeres, following their disassembly caused by centromere DNA replication (Kitamura et al., 2007), Stu2 signals had often appeared at KTs (data not shown). KTs were then caught on the lateral side of a MT extending from a spindle pole (as seen at 45 sec in this figure). In some cells, it seemed that Stu2 was subsequently transported from the KT along the KT-associated MT towards its plus end (as seen 55-75 sec in this figure). Upon arrival of the Stu2 at the MT end, the MT showed conversion from shrinkage to re-growth, i.e. the MT was rescued (75-90 sec). We observed 3 discernible events of Stu2 transport from the KT towards the MT plus end in physiological conditions and in all of these, Stu2 arrival at the MT ends led to MT rescue. This is consistent with a pivotal role of Stu2 in KT-dependent MT rescue in physiological conditions, as suggested with the engineered assay.

Discussion: The results in this figure and Figs S1D suggest that the mechanisms for KT-dependent microtubule rescue, which we found using the engineered assay (see Fig 1A), are relevant to physiological conditions.

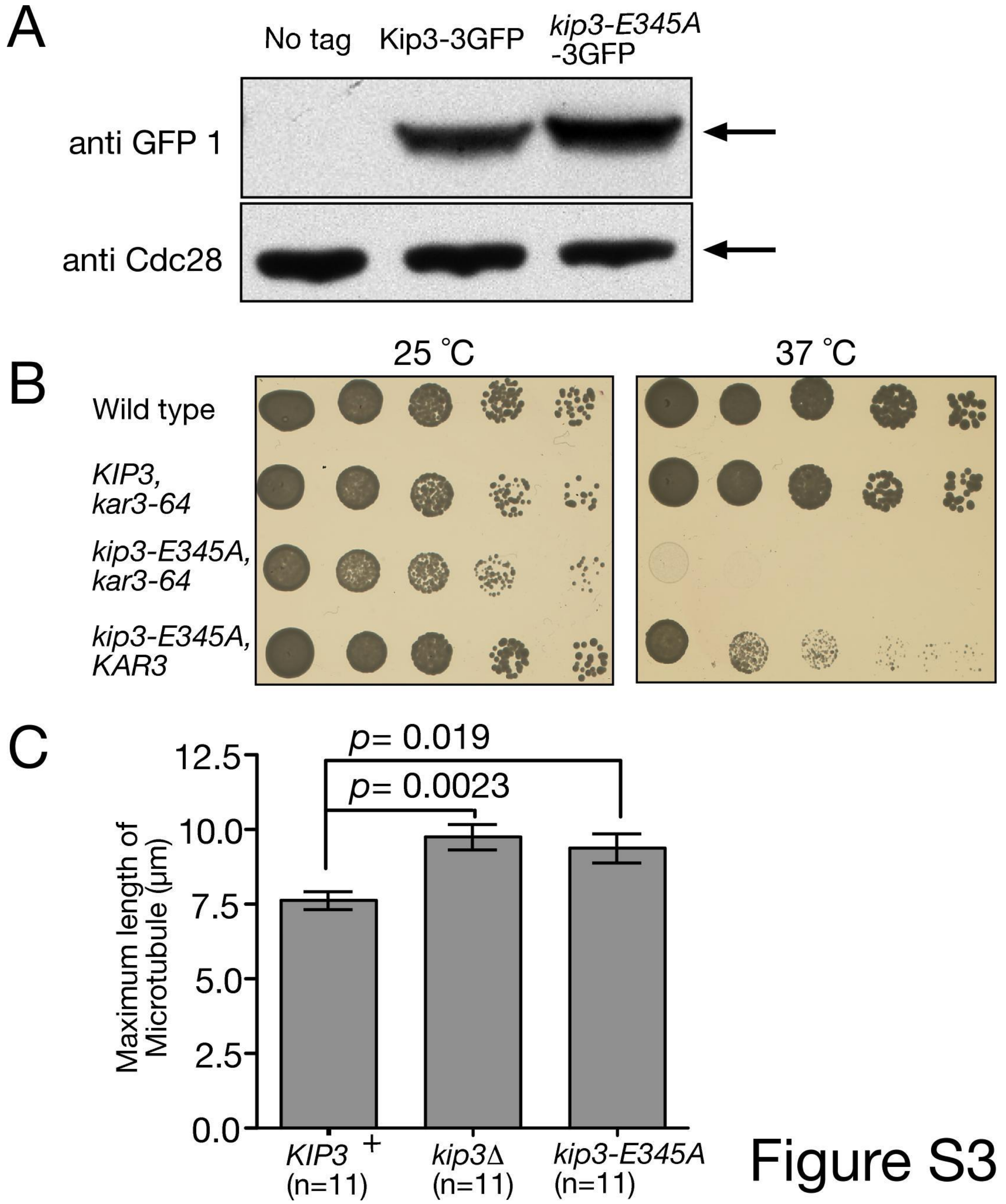
A
No tag
Kip3-3GFP
kip3-E345A
-3GFP
anti GFP 1
anti Cdc28
B
25 °C
37 °C
Wild type
KIP3,
kar3-64
kip3-E345A,
kar3-64
kip3-E345A,
KAR3
C
p= 0.019
p= 0.0023
Maximum length of
Microtubule (µm)
12.5
10.0
7.5
5.0
2.5
0.0
KIP3 +
(n=11)
kip3Δ
(n=11)
kip3-E345A
(n=11)

Figure S3

Figure S3 (associated with Figure 4)

(A) Western blot analysis of *kip3-E345A* and wild-type *KIP3* expression.
Yeast whole cell extract was prepared from strains containing either *kip3-E345A-3×GFP* (T7707) or *KIP3-3×GFP* (T3918). For control, a strain containing no GFP tag (K699) was used. As a loading control, the same samples were analyzed using anti Cdc28 antibody.

(B) *kip3-E345A* is synthetically lethal with a *kar3-64* mutant.
Growth of wild-type cells (K699) as well as cells containing either only *kip3-E345A* mutation (T7349) or only *kar3-64* mutation (T3932) were compared with cells containing both *kip3-E345A* and *kar3-64* mutation (T8096). 10-fold serial dilutions of the cells were spotted on YPD plates and incubated at 25° C and at 37° C for 48 hours. Note that *kar3-64* is a temperature sensitive mutant, which is defective at 37°C (Cottingham et al., 1999).
Results: Similarly to *kip3*Δ (Cottingham et al., 1999), *kip3-E345A* was synthetically lethal with *kar3-64* (Kar3 is a kinesin-14 family member) at the restrictive temperature (37° C) for the *kar3* mutant.

(C) *kip3*Δ and *kip3-E345A* show longer MTs than wild-type *KIP3*.
Graph showing the maximum MT length (mean ± standard error of the mean) in *KIP3*$^{+}$ (T3680), *kip3*Δ (T3776) and *kip3-E345A* (T8657) cells (see detailed genotypes in the Fig 4D legend). Cells were treated and images acquired as in Fig 4D. The maximum length of a *CEN3*-associated MT was measured.
Results: *kip3-E345A* showed longer nuclear MTs than wild-type *KIP3,* as did *kip3*Δ (Miller et al., 1998).

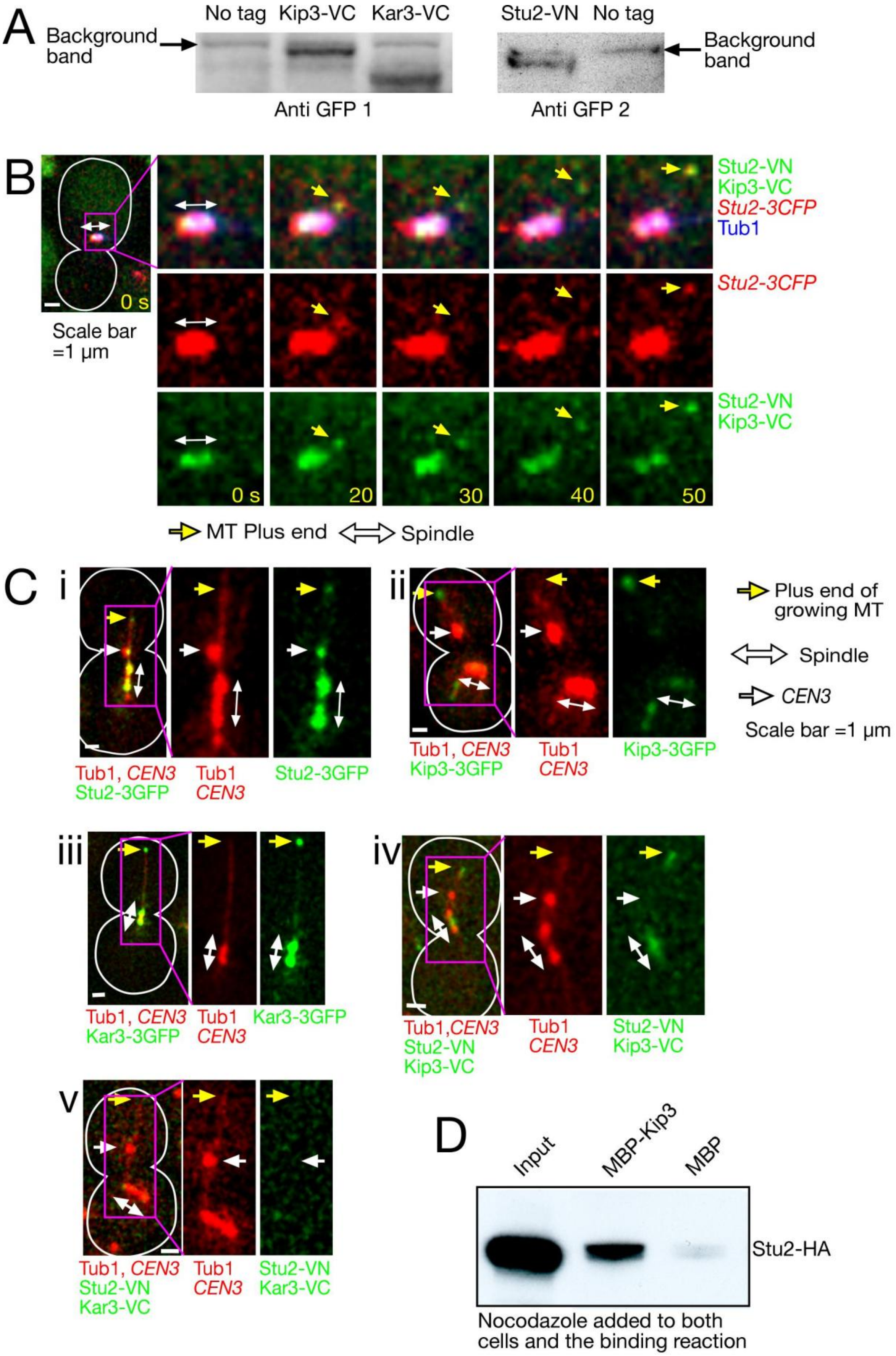
A
No tag
Kip3-VC
Kar3-VC
Stu2-VN
No tag
Background band
Anti GFP 1
Anti GFP 2
B
Stu2-VN
Kip3-VC
Stu2-3CFP
Tub1
Scale bar =1 µm
0 s
20
30
40
50
MT Plus end
Spindle
C
i
ii
iii
iv
v
Plus end of growing MT
Spindle
CEN3
Scale bar =1 µm
Tub1, CEN3
Stu2-3GFP
Kip3-3GFP
Kar3-3GFP
Tub1,CEN3
Stu2-VN
Kip3-VC
Kar3-VC
D
Input
MBP-Kip3
MBP
Stu2-HA
Nocodazole added to both cells and the binding reaction

Figure S4

Figure S4 (associated with Figure 5)

(A) Western blot analysis of *KAR3-VC*, *KIP3-VC* and *STU2-VN* expression.
Yeast whole cell extract was prepared from strains containing *KAR3-VC* (T6747), *KIP3-VC* (T6451) and *STU2-VN* (T6586). Kar3-VC and Kip3-VC were detected by using a monoclonal anti-GFP antibody (a mixture of clones 7.1 and 13.1; Roche; shown as anti-GFP1). Stu2-VN was detected by using an N-terminal specific anti-GFP antibody (Sigma; shown as anti-GFP2). Whole cell extract of K700 was used as a negative (No tag) control.
Results: The expression of Kar3-VC, Kip3-VC and Stu2-VN was confirmed by the western blot. Kar3-VC and Kip3-VC were expressed in a similar level.

(B) Venus could be formed rapidly to emit its signal when Stu2-VN and Kip3-VC first became closely associated.
Time-lapse images of *STU2-3×CFP/STU2-VN mCherry−TUB1 KIP3-VC* diploid cells (T7093) showing an example of a BiFC signal due to close association between Stu2-VN and Kip3-VC at the plus end of a newly formed microtubule. The cells were treated in the same way as Fig 1C. Images were collected every 10 sec using mCherry (Tubulin), CFP (Stu2) and YFP (BiFC signals from Stu2-VN plus Kip3-VC) channels. Scale bar, 1 µm.
Results: We tested whether Venus could be formed rapidly to emit its signal when Stu2-VN and Kip3-VC first became closely associated at the MT plus end. This seemed to be indeed the case as Venus signals appeared at the MT end, almost immediately after MTs started growing from a spindle pole.
Discussion: We interpreted this result as Venus being formed at the MT end by Stu2-VN and Kip3-VC molecules brought there separately. However, an alternative possibility is that Stu2-VN and Kip3-VC became closely associated elsewhere, in advance, and were brought together to the MT end, only becoming visible due to their accumulation. In this alternative case, we would expect that Venus signals should appear at any sites where Stu2 molecules are present and are rapidly turned over, for example at a free *CEN* in the engineered assay (see Fig 1A) where Stu2-GFP signals are intense and rapidly turning over ($t_{1/2}$ = 20-30 sec), as determined by fluorescence recovery after photo-bleaching (data not shown). However, when Stu2-VN and Kip3-VC were expressed in the same cell, Venus signals were not detected at free *CEN3*. Thus it is unlikely that Venus signals at the MT end in this figure were due to pre-formation of Venus from Stu2-VN and Kip3-VC elsewhere.

(C) *STU2-VN KIP3-VC*, but not *STU2-VN KAR3-VC*, gives BiFC signals at the MT end.
Images of *STU2-3×GFP* (i; T3680), *KIP3-3×GFP* (ii; T3981), *KAR3-3×GFP* (iii; T3733), *STU2-VN KIP3-VC* (iv; T6736) and *STU2-VN KAR3-VC* (v; T7029) cells with P_{MET3}-*CDC20* P_{GAL}-*CEN3-tetOs TetR-3×CFP CFP-TUB1.* The images show localisation of Stu2, Kip3 and Kar3 (i, ii and iii, respectively). Stu2-VN and Kip3-VC show close association at the plus end of MTs (iv), while no Venus signal was visible in cells with Stu2-VN plus Kar3-VC (v). The cells were treated as Fig 1C. Images were acquired using CFP (*CEN3,* tubulin), and GFP/YFP (Stu2, Kip3, Kar3 and BiFC) channels. Scale bar, 1 µm.
Results: A BiFC signal is generated only when two proteins are very closely associated (Kerppola, 2008). In fact, although both Stu2 and a kinesin-14 family member Kar3 localized at the MT plus ends (Fig S4C i, iii), Stu2-VN and Kar3-VC did not give Venus signals (Fig S4C v), in contrast to Stu2-VN and Kip3-VC that generated Venus signals at the MT end (Fig S4C i, ii and iv). The results confirm that Venus signals are not promiscuously generated from two proteins co-localizing with light-microscopy resolution.

(D) Stu2 binds Kip3 *in vitro* in the presence of nocodazole.
STU2-HA cells were treated with nocodazole (15 µg/ml). The binding reaction was performed as in Fig 5D, but in the presence of nocodazole. Stu2-HA bound to either MBP-Kip3 or MBP alone was detected by Western blot. Input is 1/100 of reaction. Note that we have confirmed that, with this nocodazole concentration, no MTs were detected in cells with *GFB-TUB1* (data not shown).
Results: Although both Stu2 and Kip3 bind MTs, the Stu2 interaction with Kip3 was not merely an indirect association mediated by MTs because it was detected after MTs were depolymerised with addition of nocodazole to both cell culture and the binding reaction. Nonetheless this result does not exclude the possibility that Stu2-Kip3 interaction is indirect and mediated by a third protein (other than MTs).

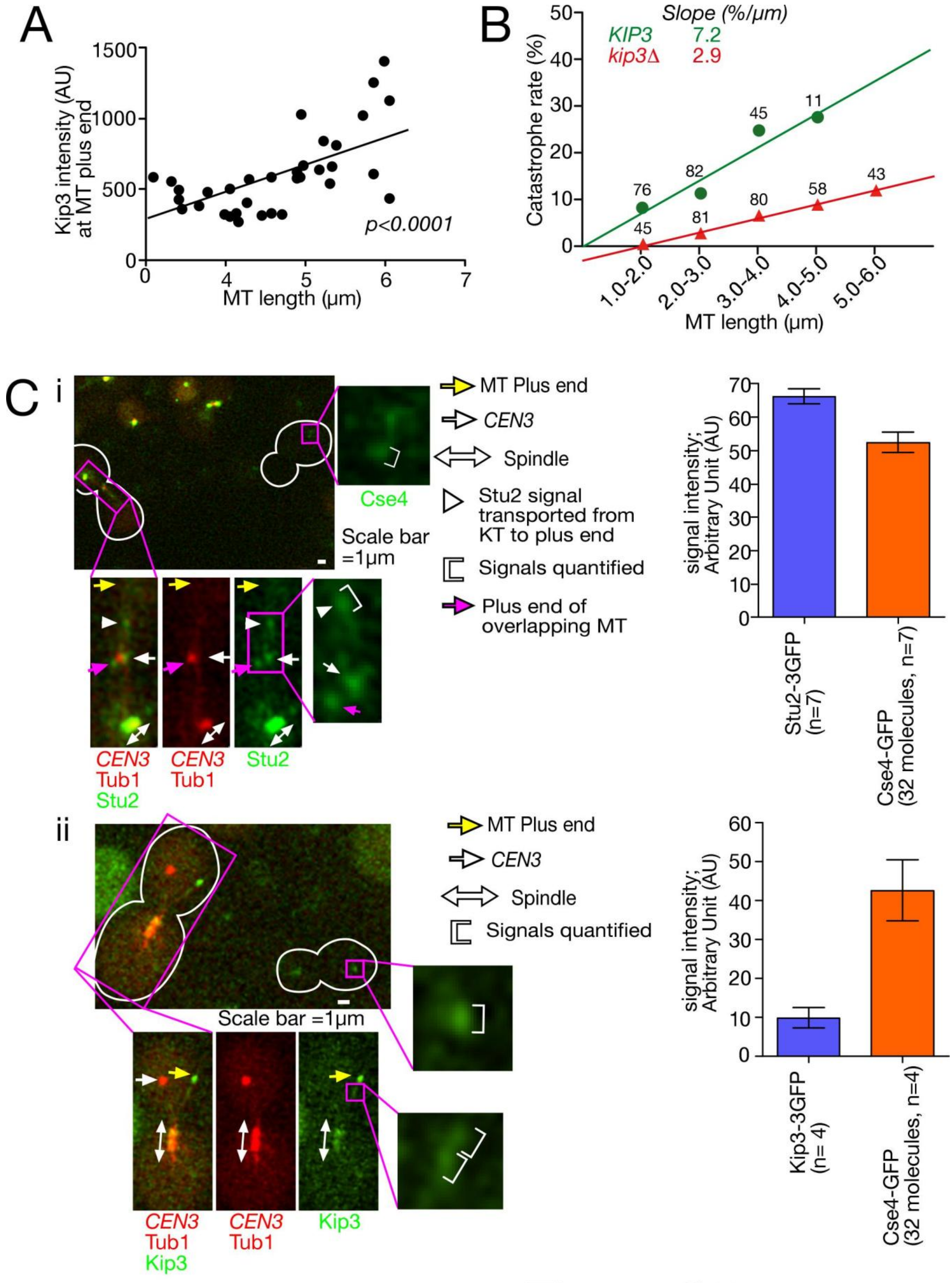
A
Kip3 intensity (AU) at MT plus end
MT length (µm)
p<0.0001
B
Catastrophe rate (%)
Slope (%/µm)
KIP3 7.2
kip3Δ 2.9
1.0-2.0
2.0-3.0
3.0-4.0
4.0-5.0
5.0-6.0
MT length (µm)
C
i
Cse4
MT Plus end
CEN3
Spindle
Stu2 signal transported from KT to plus end
Signals quantified
Plus end of overlapping MT
Scale bar =1µm
CEN3 Tub1 Stu2
CEN3 Tub1
Stu2
signal intensity; Arbitrary Unit (AU)
Stu2-3GFP (n=7)
Cse4-GFP (32 molecules, n=7)
ii
MT Plus end
CEN3
Spindle
Signals quantified
Scale bar =1µm
CEN3 Tub1 Kip3
CEN3 Tub1
Kip3
signal intensity; Arbitrary Unit (AU)
Kip3-3GFP (n= 4)
Cse4-GFP (32 molecules, n=4)

Figure S5

Figure S5 (associated with Figure 6)

(A) A larger amount of Kip3 is present at the ends of longer MTs.
The graph shows the maximum length of MTs and Kip3 intensity at the MT plus ends just before catastrophe happens. The line shows liner regression. The images acquired for Fig 6A were analyzed for this purpose. AU: arbitrary unit.
Results: Previous results *in vitro* show that Kip3 molecules are collected by a MT lateral surface then move along the MT to its plus end and accumulate there, leading to MT catastrophe (Varga et al., 2006; Varga et al., 2009). If this also occurs *in vivo*, we expect that the amount of Kip3 would be higher at the ends of longer MTs. The graph indeed shows a positive correlation between the two values ($p < 0.0001$). Moreover, we could visualize Kip3 signals emerging on the MT lateral surface and subsequently moving along a MT *in vivo* (see Fig 5A i; see also Fig 4 in (Varga et al., 2006), consistent with the idea that Kip3 is collected by the MT lateral surface.

(B) Longer MTs show a higher frequency of MT catastrophe and this correlation is partly dependent on Kip3.
Graph showing catastrophe frequency (%) plotted against MT length. *KIP3*$^+$ wild-type (T3531) and *kip3*Δ (T2834) cells with P_{MET3}-*CDC20* P_{GAL}-*CEN3-tetOs TetR-GFP YFP-TUB1* were treated as in Fig 1C, but with *CEN3* always active (i.e. incubated continuously in glucose-containing media). Images were collected every 15 sec for GFP and YFP in the same channel. The MTs emerging from a spindle pole during the first 10 min of imaging were analyzed until catastrophe happened (31 and 25 MTs were analyzed in T3531 and T2834, respectively). The y-axis shows the percentage of time points at which catastrophe occurred for MTs when MT length was within the ranges 1-2, 2-3, 3-4 μm etc (x-axis); out of the time points (their number is shown above each plot) at which MT length was within each range. Linear regressions and values of their slopes are shown.
Results: The previous *in vitro* data suggested that Kip3 accumulation at the MT end leads to MT depolymerization/catastrophe with greater frequency the longer the MTs (Varga et al., 2006; Varga et al., 2009). We tested whether this is also the case *in vivo*. As seen in this graph, both *KIP3*$^+$ wild-type and *kip3*Δ cells showed an increase of MT catastrophe frequency as MTs became longer. Compared with *kip3*Δ, *KIP3*$^+$ wild-type showed a greater frequency of MT catastrophes for longer MTs, suggesting that the MT length-dependent effect is partly dependent on Kip3.

(C) Estimating the number of Stu2 and Kip3 molecules in a single transport.
Stu2-3×GFP (i, T3680) and Kip3-3×GFP (ii, T3981) signal intensity during their transport along a microtubule in cells containing P_{MET3}-*CDC20* P_{GAL}-*CEN3-tetOs TetR-3×CFP CFP-TUB1* was compared with the Cse4-GFP (T6799) signal intensity during metaphase or telophase. T3680 and T3981 cells were treated as in Fig 1C and were mixed with an asynchronous culture of T6799 cells before imaging. Graphs on left show arbitrary values of signal intensity (mean ± standard error of the mean). Scale bar, 1 μm.
Results: Cse4-GFP shows a bi-lobular localization pattern during metaphase and one lobe should contain 32 GFP molecules (Furuyama and Biggins, 2007; Joglekar et al., 2006). The Cse4-GFP signal at a spindle pole in telophase should also contain 32 GFP molecules. We quantified the intensity of Stu2-3×GFP and Kip3-3×GFP signals in a single transport and compared their intensity with the intensity of the Cse4-GFP signal (between the cells in the same microscopy field). From this comparison, we estimated that about 10-15 Stu2 molecules and 2-3 Kip3 molecules moved along a MT in a single transport event.

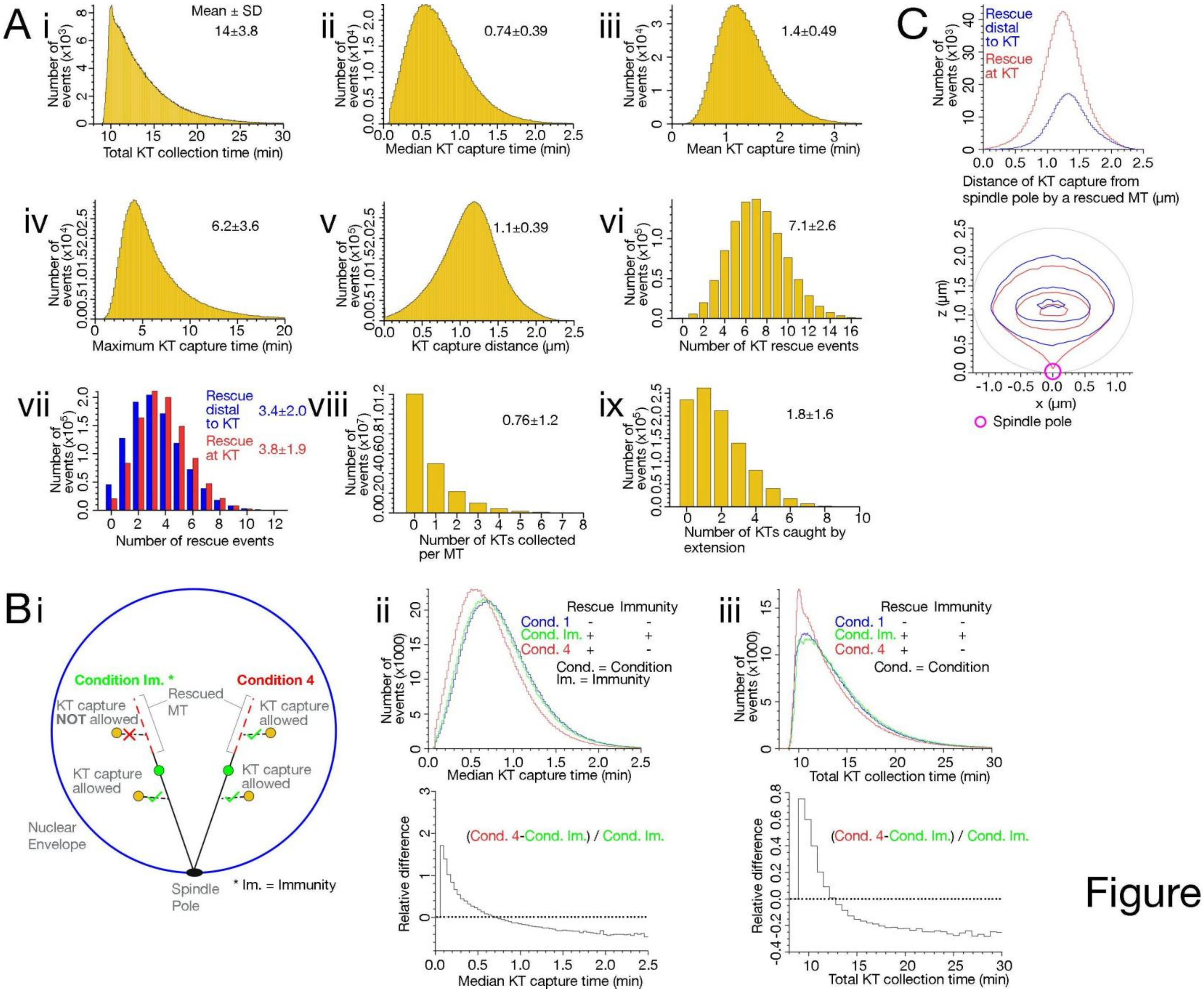
A
Mean ± SD
14±3.8
Total KT collection time (min)
0.74±0.39
Median KT capture time (min)
1.4±0.49
Mean KT capture time (min)
6.2±3.6
Maximum KT capture time (min)
1.1±0.39
KT capture distance (μm)
7.1±2.6
Number of KT rescue events
Rescue distal to KT 3.4±2.0
Rescue at KT 3.8±1.9
Number of rescue events
0.76±1.2
Number of KTs collected per MT
1.8±1.6
Number of KTs caught by extension
Number of events
C
Rescue distal to KT
Rescue at KT
Distance of KT capture from spindle pole by a rescued MT (μm)
z (μm)
x (μm)
Spindle pole
B
Condition Im. *
Condition 4
Rescued MT
KT capture NOT allowed
KT capture allowed
Nuclear Envelope
Spindle Pole
* Im. = Immunity
Rescue Immunity
Cond. 1
Cond. Im.
Cond. 4
Cond. = Condition
Im. = Immunity
Number of events (x1000)
Median KT capture time (min)
Total KT collection time (min)
Relative difference
(Cond. 4-Cond. Im.) / Cond. Im.

Figure S6

Figure S6 (associated with Figure 7)

(A) Various evaluations of KT-MT interactions from simulations.
One million simulations were run in 'wild-type' conditions, i.e. in the presence of MT rescue both at, and distal to the KT (condition 4 in Fig 7C). The value at the top right corner in each graph shows mean ± SD. Total KT collection time was defined in the text and evaluated in individual simulations (i). KT capture time was also defined in the text and median (ii), mean (iii) and maximum (iv) KT capture time was evaluated in individual simulations. KT capture distance (from a spindle pole) was recorded upon encounter of each KT with a spindle-pole MT (v) (along any region of a MT, in contrast to Figs 7D and S6C, in which we analyzed KT encounters only with extended MT regions following rescue). The number of MT rescue events in individual simulations is plotted together (vi) or separately (vii) for rescue at the KT and distal to the KT. The number of KTs collected per MT was evaluated for individual MTs (viii). The number of KTs caught by rescued and extended MTs (along the extended MT region) was evaluated in individual simulations (ix). The binning along the x-axis was 0.04 min (i, iii, iv), 0.02 min (ii) and 0.015 μm (v).

(B) The effects of KT-dependent MT rescue on overall KT collection are mainly due to capture of other KTs by the extended MT region following MT rescue.
KT-dependent MT rescue causes a number of changes in MT dynamics, leading to capture of other KTs. For example, MTs extend and capture other KTs that localize further away from a spindle pole than the point of MT rescue (e.g. capture of *CEN13* and *CEN3* in Fig 7B). On the other hand, the MT region between the MT rescue point and a spindle pole remains for a longer period following KT-dependent MT rescue, and thus also captures other KTs (e.g. capture of *CEN16* in Fig 7B). Which effect contributes more to shortening KT collection time, as shown in Fig 7C?
(i) To test this, we imposed settings that disallowed capture of KTs by the MT region extended following MT rescue, but still allowed capture using the MT region between the rescue point and a spindle pole; we termed this 'immunity'.
(ii, iii) We ran one million simulations with this 'immunity' (condition Im.) and compared the outcomes with those from simulations having no 'immunity', in the presence and absence (condition 4 and 1 in Fig 7C, respectively) of KT-dependent MT rescue (at the KT and distal to the KT).
Results: The distribution of median KT capture time and total KT collection time with 'immunity' was very similar to that without MT rescue (condition 1). The relative difference was also similar between Fig 7C i (bottom) and Fig S6B ii (bottom), and between Fig 7C ii (bottom) and Fig S6B iii (bottom). We conclude that the effect of KT-dependent MT rescue on overall KT collection is mainly attributed to capture of other KTs by the extended MT region following MT rescue.

(C) MT rescue distal to the KT is particularly useful for collecting KTs that have drifted further away from a spindle pole.
Graph (top) shows distribution of the distance form a spindle pole of KT capture by the extended region of MTs, following rescue at the KT (red) and rescue distal to the KT (blue), in 'wild-type' conditions (condition 4 in Fig 7C). The binning along the x-axis was 0.03 μm. Contour map (bottom) shows the distribution of KT capture position density. Contour levels are 0.95, 0.6 and 0.1 (inside to outside) of the maximum value. Capture counts were calculated as cumulative values along the y-axis, projected to the x-z plane.

Supplemental Experimental Procedures

Yeast genetics and molecular biology

The background of yeast strains (W303) and methods for yeast culture and α-factor treatment were as described previously (Amberg et al., 2005; Tanaka et al., 2007). Cells were cultured at 25 ºC in YP medium containing 2 % glucose, unless otherwise stated. Constructs of *P_{GAL}-CEN3-tetOs* (Tanaka et al., 2005), *TetR-3×CFP* (Bressan et al., 2004), *P_{MET3}-CDC20* (Uhlmann et al., 2000) and *TetR-GFP* (Michaelis et al., 1997) were as described previously. To make *kip1Δ*, *cin8Δ*, *kip2Δ* and *kip3Δ*, whole open reading frames of the relevant genes were replaced with *KanMX4* and *HIS3* gene, using a one-step PCR method (Amberg et al., 2005); *kip1Δ*, *cin8Δ*, and *kip3Δ* were obtained from EUROSCARF, Frankfurt, Germany.

KIP1, CIN8, KIP2, KIP3, *STU2*, *BIK1*, *BIM1*, *KAR3, NDC80* and *MTW1* were tagged at their C-termini at their original gene loci by a one-step PCR method (Maekawa et al., 2003; Tanaka et al., 2005), using *3×GFP-KanMX6* (pSM1023; (Maekawa et al., 2003), *3×CFP-HIS3* (pT769), *4×mCherry-NatMX6* (pT909) and *6×HA-HIS3* (a gift from Kim Nasmyth lab, University of Oxford) cassettes as PCR templates. pT909 was constructed by multiplying the *mCherry* gene in pKS391 (Snaith et al., 2005). To construct *STU2ΔTOG1-3×GFP*, PCR was carried out with the primers flanking the *TOG1* domain but in a reverse manner using *STU2 (wild-type)-3×GFP* as a PCR template. The *STU2ΔTOG1-3×GFP* plasmid was completed by circularizing the PCR product by ligation and was integrated at an auxotroph locus as a single copy.

To construct *kip3-E345A* mutant, the mutation was introduced to *KIP3* gene cloned in pGEM T-easy plasmid, using the QuickChange II site-directed mutagenesis kit (Strategene). *KIP3::URA3* (*URA3* inserted within *KIP3* gene) was replaced with the mutated *kip3*, using 5-fluoroorotic acid selection. For the BiFC assay, *KIP3, STU2* and *KAR3* were tagged at their C-termini at their original gene loci, using *VN-TRP1* and *VC-HIS3MX3* cassettes, containing N-terminal and C-terminal halves of the gene for Venus fluorescent protein (Sung and Huh, 2007). *GFP-TUB1* (Straight et al., 1997), *CFP-TUB1* (Janke et al., 2002), *YFP-TUB1* (pDH20, obtained from Yeast Resource Centre, Seattle, USA) and *mCherry-TUB1* (constructed for this study) plasmids were integrated at auxotroph marker loci. Strains with the tagged genes grew normally at temperatures used in this study.

Live-cell imaging

The procedures for time-lapse fluorescence microscopy were described previously (Kitamura et al., 2007; Tanaka et al., 2007). Time-lapse images were collected at 25 ºC (ambient temperature). For image acquisition, we used a DeltaVision RT microscope (Applied Precision), UPlanSApo 100× objective lens (Olympus; NA 1.40), a CoolSnap HQ CCD camera (Photometrics) and SoftWoRx software (Applied Precision). We acquired 5-9 (0.7 μm apart) z-sections, which were subsequently deconvoluted and analyzed with SoftWoRx and Volocity (Improvision) software. For figures, z stacks were projected to two-dimensional images. GFP signals were discriminated from YFP, using the JP3 filter set (Chroma). CFP, YFP (or GFP) and mCherry signals were discriminated with the 89006 ET filter set (Chroma). GFP and YFP signals were acquired together, using the YFP channel of the 89006 ET filter set.

Analyzing dynamics of kinetochores, microtubules and associated proteins

To evaluate the length of MTs and position of centromeres, we took account of the distance along the z-axis as well as distance on each z plane. To evaluate the period of MT pausing at *CEN3* (Fig 1D, S1C), *CEN3* motion was fit to linear regression while it was at the MT end, and the period with MT shrinkage less than 1.0 μm/min and with no growth was determined; see example in the graph of Fig 2A iv. Statistical analyses were carried out with the a) a chi-square test (Figs 2A ii, 2B ii, S1D), b) a paired *t*-test (Fig S1B), c) Fisher's exact test (Figs 3B iii, 4B, 4C ii, 4D i), d) an unpaired *t*-test (Figs 4D ii, S3C) and e) *p* value based on correlation (Fig S5A). The null hypotheses in these tests were that the samples were collected randomly and independently, from the same population (a to d) or from the population with no correlation between X and Y (e). All *p* values were two-tailed, and the null hypotheses were reasonably discarded when *p* values were < 0.05.

Discerning the 'end-on attachment': Only after *CEN3* was pulled towards a spindle pole as the MT shrinks (end-on pulling), we could recognize the KT-MT attachment as 'end-on attachment', because only after end-on pulling started we could confirm that the KT on *CEN3* was coupled or tethered properly at the MT end. Likewise, if a MT end had reached the KT but end-on pulling had not yet begun, we did not yet know whether end-on attachment had been formed or was still in an intermediate state between lateral and end-on attachment.

Protein pulldown assay and Western blots

MBP-Kip3 or MBP in pLous3 (a gift from Jim Naismith lab, University of St Andrews) was expressed in *E. coli* and bound to amylose beads approximately at 0.2 µg/µl. 5 µl of beads was incubated for 45 mins at 4˚C with an extract of yeast cells expressing Stu2-6×HA from the endogenous promoter. Yeast extract was prepared by bead beating in a buffer containing 50mM HEPES pH7.6, 150mM NaCl, 0.05% Tween20, 10mM β mercaptoethanol, protease inhibitors (Calbiochem) and phosphatase inhibitors (PhosStop, Roche). After the binding reaction, beads were washed, by resuspension and centrifugation, once in binding buffer containing 500mM NaCl, then a further twice in binding buffer before the beads were eluted by heating at 100˚C in SDS gel loading buffer. Stu2-6×HA was detected on Western blots using 12CA5 anti-HA antibody (Fig 5D). Where nocodazole was used (Fig S4D), it was added to yeast cells for 2 hrs prior to harvesting and to binding reactions at 10 µg/ml. To detect Kip3-3×GFP, Kar3-VC, Kip3-VC and Stu2-VN proteins (Figs S3A, S4A), yeast cell extracts were prepared as above and Western blots were carried out using anti-GFP antibodies (G1544, Sigma for Stu2-VN; clones 7.1/13.1, Roche for others).

Computer simulation of kinetochore interaction with microtubules

Parameter	Symbol	Value	Source of the value
Time step	Δt	0.01 min	A reasonably small value was chosen
Radius of the nucleus	R_{nuc}	1.25 µm	This study (visualization of the nuclear envelope)
MT growth speed	v_{gro}	1.5 µm min^{-1}	Fig 3b, Tanaka et al. 2005
MT shrinkage speed	v_{shr}	2.8 µm min^{-1}	Fig 3b, Tanaka et al.

			2005
MT catastrophe rate	K_{cat}	0.6 min^{-1}	This study
MT nucleation rate	K_{nuc}	0.5 min^{-1}	Based on Fig S1E etc, Kitamura et al., 2010
MT beaming factor	β	0.7	Based on Fig S1E etc, Kitamura et al., 2010
Diffusion constant	D	0.1 $\mu m^2\ min^{-1}$	Fig S1, Kitamura et al., 2007
KT lateral sliding speed	v_{lat}	1 $\mu m\ min^{-1}$	Fig 7C, Kitamura et al. 2007
KT end-on pulling speed	v_{pul}	1.7 $\mu m\ min^{-1}$	Fig 2D, Tanaka et al. 2007 & Fig 7C, Kitamura et al. 2007
KT slow end-on pulling speed	v_{spul}	0.35 $\mu m\ min^{-1}$	This study
KT rescue delay	t_d	8 sec	This study
Stu2 sending rate	K_{stu2}	0.3 min^{-1}	This study
Stu2 speed	V_{stu2}	2.1 $\mu m\ min^{-1}$	Fig S9, Tanaka et al. 2005 & this study
KT capture radius	R_{KT}	0.4 μm	Fig 6A, S6A Kitamura et al., 2010
KT capture speed	v_{cap}	5 $\mu m\ min^{-1}$	Fig S1C, Kitamura et al., 2010
Probability of MT rescue at the KT	P_{res}	0.6	Fig 4B, Tanaka et al., 2007
KT reassembly position	R_{ass}	1.4 ± 0.2 μm (mean ± SD)	Fig 1, Kitamura et al., 2007

List 1. Parameters used in the computer model of KT-MT interaction. Values of parameters were obtained from our previous studies (Kitamura et al., 2007; Kitamura et al., 2010; Tanaka et al., 2007; Tanaka et al., 2005) or newly evaluated in this study.

Centromeres	Replication timing (min)
CEN2	0
CEN3	1.53
CEN13	1.65
CEN10	2.66
CEN4	2.89
CEN16	3.46
CEN5	3.49
CEN7	3.77
CEN1	3.83
CEN9	3.91
CEN12	4.05
CEN11	4.40
CEN15	5.22
CEN6	6.49
CEN14	6.54
CEN8	7.49

List 2. Replication timing of centromeres relative to *CEN2* that replicates earliest. The values were obtained from (Yabuki et al., 2002).

We created a computer model and carried out simulation of the initial KT-MT interaction (Fig 7A), based on configuration in the physiological conditions (Kitamura et al., 2007); i. e. MTs extend from a single spindle pole, capture KTs and bring them back to the vicinity of the spindle pole. In this simulation, we used the parameters shown in List 1. The values of the majority of the parameters were defined in our previous studies of live-cell imaging and electron tomography (Kitamura et al., 2007; Kitamura et al., 2010; Tanaka et al., 2007; Tanaka et al., 2005), and unknown parameters were measured in the current study. Whenever possible, the parameter values were estimated in the physiological experimental conditions (Kitamura et al., 2007). Only when it was difficult to estimate them in physiological conditions, did we use an engineered condition (see Fig 1A).

The model was computed as a discrete simulation of a series of events on a constant time step Δt. All objects (MTs, KTs and Stu2) were located in a 3-dimentional space in a Cartesian reference frame with the *z*-axis pointing “up”. The nucleus was represented by a sphere of radius R_{nuc}, centred along the *z*-axis at the distance of R_{nuc} from the origin. A spindle pole was located at the origin. Each MT was a line segment extending into the nucleus from the spindle pole. Each KT was a point inside the nucleus. A group of Stu2 proteins in a single transport event was a point on a MT.

MTs could grow and shrink with speed v_{gro} and v_{shr}, respectively. A catastrophe (conversion from growth to shrinkage) could happen randomly at a rate of K_{cat}, calculated only over the growth stage. When a growing MT hit the nuclear envelope, it started to shrink. When an empty MT shrank to the spindle pole, it could start growing at a certain nucleation rate K_{nuc}, unless there was an excessive KT waiting at the spindle pole (see below), in which case the MT captured the KT and they showed no further change. The value K_{nuc} was determined so that the 5-10 long MTs (> 0.7 μm) appeared during KT collection, as estimated from electron tomography images in early S phase (e.g. Fig S1E in Kitamura et al., 2007). When Stu2 moving along a MT reached its distal end, a shrinking MT was rescued i.e. shrinkage was converted into growth. When the distal end of a shrinking MT caught up with a laterally sliding KT, it could be rescued with probability P_{res}. Otherwise end-on pulling was commenced; MTs were not rescued during end-on pulling (Tanaka et al., 2007).

MT direction was represented by two angles, the azimuth angle, φ, and the zenith angle (measured from the *z*-axis), θ. MTs grew and re-grew in random directions with angular distribution concentrated towards the centre of the nucleus. We assumed the MTs did not change direction as they grew, as such changes were relatively small in live cell imaging (data not shown). To simulate this situation, we initially generated an isotropic direction, $\varphi = R[0, 2\pi]$ and $\cos\theta = R[0, 1]$, where R[*a*, *b*] was a random number uniformly distributed between *a* and *b*. We transformed the zenith angle with a “beaming” factor, β:

$$\cos\theta' = \frac{\cos\theta + \beta}{1 + \beta\cos\theta}$$

The angles φ and θ’ give the desired distribution, which is isotropic for $\beta = 0$ and becomes increasingly more concentrated towards the centre of the nucleus as $\beta \to 1$. The value for β was estimated so that MTs extending from a spindle pole formed a configuration to images of electron tomography acquired in early S phase (e.g. Fig S1E in (Kitamura et al., 2007),

Time 0 was defined as the time of replication of the first *CEN* (*CEN2*) and therefore its detachment from a spindle pole. At -10 min, 5 MTs started growing from a spindle

pole (this number was set so that 5-10 long MTs [> 0.7 μm] appeared during KT collection; see above). Each new MT became available for growth and shrinkage when replication of each centromere (List 2; Yabuki et al., 2002) caused KT disassembly and centromere detachment from a MT. One minute after this, a KT was reassembled at each detached centromere and became capable of reattaching to a MT. We assumed that KTs were reassembled at random positions at a distance defined by R_{ass} (showing Gaussian distribution) from the spindle pole.

KTs moved freely inside the nucleus following a random walk with a diffusion constant D. Once attached to a MT, a KT slid laterally towards the spindle pole or was pulled by the distal end of the MT with speed v_{lat} or v_{pul}, respectively. When a sliding KT reached the spindle pole, it was transferred to a waiting pool where it remained until an empty MT shrank to zero. Then, the KT was caught at the end of this MT and the 'arrival' of KT to the spindle pole was completed, after which no further change occurred to such KT and MT. The same happened immediately when an end-on pulled KT reached the spindle pole.

Before centromere DNA replication caused KT disassembly and after a reassembled KT was transported to the vicinity of the spindle pole, the relevant KT stayed in the vicinity of a spindle pole. In this circumstance, KTs were tethered with the plus ends of short MTs (< 300 nm), which however were considered to have zero length for simplicity in simulation.

A KT, which was sliding on a MT or waiting for end-on establishment at the spindle pole, could send Stu2 along the MT at a rate of K_{stu2}. Stu2 moved along the MT away from a spindle pole at a speed of v_{stu2}. The arrival of Stu2 at the MT plus end led to rescue of the MT (rescue distal to the KT). When the MT plus end caught up with a KT, which was associated on the lateral side of this MT, MT rescue (rescue at the KT) occurred with probability of P_{res}, following a delay period of t_d, during which the KT was pulled by slow-speed "end-on pulling" (speed v_{spul}) (discussed as MT pausing in text). The duration of the delay was estimated based on the duration of transient co-localization of the Dam1 complex with *CEN* in physiological conditions (see Kitamura et al., 2007).

In the condition lacking MT rescue at the KT, end-on attachment was established (P_{res}=0) and the KT was pulled towards a spindle pole at a speed of v_{pul} (without prior 'pausing'). Here we did not assume KT detachment occurred from a MT, although the *stu2* hypomorphic mutant, which was defective in MT rescue at the KT, showed such detachment in 16 % of cases (Fig 2A ii). This is because our aim in the simulation was to find the benefit of MT rescue at the KT, independent of the prevention of the KT detachment.

Although K_{stu2} and K_{cat} (see above) might change depending on the position of the KT (see Discussion) and MT length (Fig S5B; Varga et al., 2006) respectively, we set them as constants for simplicity. Accordingly, the approximate values of K_{stu2} and K_{cat} were estimated, based on our measurement in the engineered assay system (see Fig 1A) and in physiological condition (Kitamura et al., 2007), respectively (note that it was hard to estimate K_{stu2} in physiological conditions).

The interaction between KT-generated and spindle-pole MTs was simplified by assuming a certain capture radius, R_{KT}, around each KT. If a KT was found at a distance R_{KT} from any part of a spindle-pole MT, the KT-derived MT connected to this spindle-pole MT by the shortest distance and brought the KT towards the spindle-pole MT, usually on its lateral side, at a speed v_{cap}, which we assumed to be the same as the depolymerization rate of a KT-derived MT (Fig S1C in Kitamura et al,

2010). Once capture was completed, the KT began sliding, which was converted to end-on pulling if end-on attachment was subsequently established. The entire simulation was completed once all 16 reassembled KTs reached the spindle pole and established end-on attachment.

If an end-on pulled KT caught up with another KT that was sliding along the same MT, in our simulation the sliding KT detached from the MT, because 1) end-on pulling was faster than sliding and 2) a single MT is thought to establish end-on attachment only for one KT (Winey et al., 1995). We assumed that the detached KT was not able to re-attach to a MT until the KT generated a MT from it at its maximum length R_{KT}; i.e. for R_{KT}/v_{gro} min (note that KT-derived MTs showed a similar growth rate to a spindle-pole MTs; Fig S1C in Kitamura et al, 2010).

The code for the simulation was written in Perl and simulations were run in a Linux environment. We ran 1,000,000 individual simulations in each condition. In Fig 7C, we tested four conditions with P_{res}= 0 or 0.6 combined with K_{stu2}=0 or 0.3. In Fig S6B, we tested the 'immunity' of rescued MTs for capture of other KTs. With this 'immunity', after a KT induced rescue of its associated MT, the rescued MT could not capture any additional KTs using the MT region acquired by the relevant MT rescue and subsequent MT extension. In Fig 7D, the position of KT capture by a spindle-pole MT was defined as the first KT contact point with a spindle-pole MT (usually on its lateral side).

Supplemental References

Amberg, D.C., Burke, D.J., and Strathern, J.N. (2005). Methods in yeast genetics (CSHL press).

Bressan, D.A., Vazquez, J., and Haber, J.E. (2004). Mating type-dependent constraints on the mobility of the left arm of yeast chromosome III. J Cell Biol *164*, 361-371.

Cottingham, F.R., Gheber, L., Miller, D.L., and Hoyt, M.A. (1999). Novel roles for saccharomyces cerevisiae mitotic spindle motors. J Cell Biol *147*, 335-350.

Furuyama, S., and Biggins, S. (2007). Centromere identity is specified by a single centromeric nucleosome in budding yeast. Proc Natl Acad Sci U S A *104*, 14706-14711.

Janke, C., Ortiz, J., Tanaka, T.U., Lechner, J., and Schiebel, E. (2002). Four new subunits of the Dam1-Duo1 complex reveal novel functions in sister kinetochore biorientation. Embo J *21*, 181-193.

Joglekar, A.P., Bouck, D.C., Molk, J.N., Bloom, K.S., and Salmon, E.D. (2006). Molecular architecture of a kinetochore-microtubule attachment site. Nat Cell Biol *8*, 581-585.

Kerppola, T.K. (2008). Bimolecular fluorescence complementation (BiFC) analysis as a probe of protein interactions in living cells. Annu Rev Biophys *37*, 465-487.

Kitamura, E., Tanaka, K., Kitamura, Y., and Tanaka, T.U. (2007). Kinetochore microtubule interaction during S phase in Saccharomyces cerevisiae. Genes Dev *21*, 3319-3330.

Kitamura, E., Tanaka, K., Komoto, S., Kitamura, Y., Antony, C., and Tanaka, T.U. (2010). Kinetochores generate microtubules with distal plus ends: their roles and limited lifetime in mitosis. Dev Cell *18*, 248-259.

Maekawa, H., Usui, T., Knop, M., and Schiebel, E. (2003). Yeast Cdk1 translocates to the plus end of cytoplasmic microtubules to regulate bud cortex interactions. Embo J *22*, 438-449.

Michaelis, C., Ciosk, R., and Nasmyth, K. (1997). Cohesins: chromosomal proteins that prevent premature separation of sister chromatids. Cell *91*, 35-45.

Miller, R.K., Heller, K.K., Frisen, L., Wallack, D.L., Loayza, D., Gammie, A.E., and Rose, M.D. (1998). The kinesin-related proteins, Kip2p and Kip3p, function differently in nuclear migration in yeast. Mol Biol Cell *9*, 2051-2068.

Snaith, H.A., Samejima, I., and Sawin, K.E. (2005). Multistep and multimode cortical anchoring of tea1p at cell tips in fission yeast. EMBO J *24*, 3690-3699.

Straight, A.F., Marshall, W.F., Sedat, J.W., and Murray, A.W. (1997). Mitosis in living budding yeast: anaphase A but no metaphase plate. Science *277*, 574-578.

Sung, M.K., and Huh, W.K. (2007). Bimolecular fluorescence complementation analysis system for in vivo detection of protein-protein interaction in Saccharomyces cerevisiae. Yeast *24*, 767-775.

Tanaka, K., Kitamura, E., Kitamura, Y., and Tanaka, T.U. (2007). Molecular mechanisms of microtubule-dependent kinetochore transport toward spindle poles. J Cell Biol *178*, 269-281.

Tanaka, K., Mukae, N., Dewar, H., van Breugel, M., James, E.K., Prescott, A.R., Antony, C., and Tanaka, T.U. (2005). Molecular mechanisms of kinetochore capture by spindle microtubules. Nature *434*, 987-994.

Uhlmann, F., Wernic, D., Poupart, M.A., Koonin, E.V., and Nasmyth, K. (2000). Cleavage of cohesin by the CD clan protease separin triggers anaphase in yeast. Cell *103*, 375-386.

Varga, V., Helenius, J., Tanaka, K., Hyman, A.A., Tanaka, T.U., and Howard, J. (2006). Yeast kinesin-8 depolymerizes microtubules in a length-dependent manner. Nat Cell Biol *8*, 957-962.

Varga, V., Leduc, C., Bormuth, V., Diez, S., and Howard, J. (2009). Kinesin-8 motors act cooperatively to mediate length-dependent microtubule depolymerization. Cell *138*, 1174-1183.

Winey, M., Mamay, C.L., O'Toole, E.T., Mastronarde, D.N., Giddings, T.H., Jr., McDonald, K.L., and McIntosh, J.R. (1995). Three-dimensional ultrastructural analysis of the Saccharomyces cerevisiae mitotic spindle. J Cell Biol *129*, 1601-1615.

Yabuki, N., Terashima, H., and Kitada, K. (2002). Mapping of early firing origins on a replication profile of budding yeast. Genes Cells *7*, 781-789.
