# Supplementary figures and images for: Kinetochore-Dependent Microtubule Rescue Ensures Their Efficient and Sustained Interactions in Early Mitosis
Source: Dev Cell. 2011 Nov 15;21(5-5):920–33. doi: 10.1016/j.devcel.2011.09.006 (PMC3277888; doi:10.1016/j.devcel.2011.09.006)

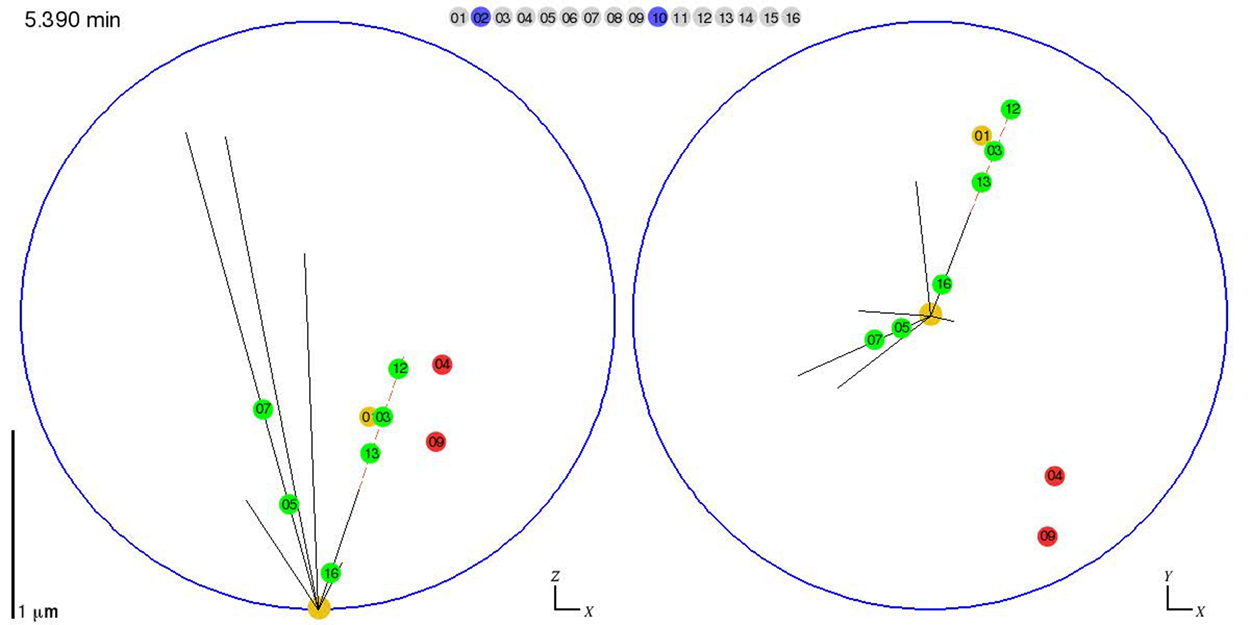

Supplement: Movie S1. An Example of Simulation in “Wild-Type” Conditions, i.e. in the Presence of MT Rescue Both at the KT and Distal to the KT (Condition 4 in Figures 7C and 7D) — The same example is also presented in Figure 7B. Projections for x-z and x-y planes are shown at the left and right, respectively. Once each CEN experiences detachment from a spindle pole (yellow dot), reattach to a MT and comes back at a spindle pole, a corresponding number at top becomes blue. [file mmc2.jpg]
